# Quantifying Lgr5-positive stem cell behaviour in the pyloric epithelium

**DOI:** 10.1038/srep21923

**Published:** 2016-02-26

**Authors:** Marc Leushacke, Nick Barker, Carmen Pin

**Affiliations:** 1 A*STAR Institute of Medical Biology, Singapore; 2 Centre for Regenerative Medicine, University of Edinburgh, Edinburgh, UK; 3 Department of Biochemistry, Yong Loo Lin School of Medicine, National University of Singapore, Singapore; 4 Gut Health and Food Safety Programme. Institute of Food Research, Norwich, UK

## Abstract

Using *in-vivo* lineage tracing data we quantified clonal expansion as well as proliferation and differentiation of the Lgr5-positive stem cell population in pyloric gastric glands. Fitting clone expansion models, we estimated that there are five effective Lgr5-positive cells able to give rise to monoclonal glands by replacing each other following a pattern of neutral drift dynamics. This analysis is instrumental to assess stem cell performance; however, stem cell proliferation is not quantified by clone expansion analysis. We identified a suitable mathematical model to quantify proliferation and differentiation of the Lgr5-positive population. As expected for populations in steady-state, the proliferation rate of the Lgr5-positive population was equal to its rate of differentiation. This rate was significantly faster than the rate at which effective cells are replaced, estimated by modelling clone expansion/contraction. This suggests that the majority of Lgr5-positive cell divisions serve to renew epithelial cells and only few result in the effective replacement of a neighbour to effect expansion to the entire gland. The application of the model under altered situations with uncoupled differentiation and proliferation was demonstrated. This methodology represents a valuable tool for quantifying stem cell performance in homeostasis and importantly for deciphering altered stem cell behaviour in disease.

The adult mammalian stomach in the mouse comprises three anatomically distinct regions: the proximal non-glandular fore-stomach, the glandular corpus and the distal glandular pylorus. The epithelial lining of the glandular stomach represents a single cell layer epithelium that is organized into multiple gastric units resembling flask-shaped pockets termed glands. Individual glands consist of a gland base, a neck and an isthmus domain connecting to a pit compartment, which opens out onto the gastric surface epithelium^1^. Limited reservoirs of adult stem cells residing within individual glands effect tissue renewal of the stomach epithelium throughout life. The precise morphological structure and turnover rate of individual gastric units may differ considerably among one another, depending on their anatomical region and cellular composition.

Multiple actively proliferating Lgr5-positive cells have been identified to reside at the base of each pyloric gland. *In vivo* lineage analysis characterized these cells as being self-renewing, multipotent adult stem cells involved in long-term renewal of the pyloric epithelium under normal homeostasis conditions^2^. Preservation of the adult stem cell pool is essential to ensure optimal tissue homeostasis throughout adulthood. A balanced homeostasis of the pyloric epithelium and the Lgr5-positive stem cell pool is predominantly achieved via neutral competition between symmetrically dividing Lgr5-positive stem cells^3^. Long term tracing studies identified such Lgr5-positive derived stem cell clones to expand laterally within the pyloric epithelium via gland fission over time^3^. In the corpus epithelium, lineage-tracing studies with chemical mutagenesis^4^ or genetic tracing from the Sox2 locus^5^ have validated the existence of multipotent stem cells. However, the broad expression pattern of Sox2 in the corpus glands precludes its qualification as a bona-fide stem cell marker. In 2013, Troy + chief cells at the gland base were identified as a reserve population of corpus stem cells that contribute to epithelial repair following major damage^6^. More recently, Mist1 expression has been determined to mark mature chief cells at the lower third of the glands as well as relatively quiescent stem cells in the gastric corpus isthmus^7^.

In the intestine, mathematical modelling of the monoclonal expansion of stem cells based on lineage tracing datasets has been instrumental in demonstrating that small intestinal epithelial stem cells are equipotent with respect to their ability to populate the entire gland, typically divide symmetrically and are replaced at random according to a neutral drift pattern^8^. Although all Lgr5-positive cells have long-term self-renewal potential, those cells in advantageous position are effective stem cells giving rise to monoclonality^9^. This positional bias leads to a clonal expansion dynamics that can be approximated by a one dimensional random walk model with absorbent boundaries^8^,^9^. Fitting these models, it has been found that only 5 to 7 of the 16 Lgr5-positive stem cells are able to effectively give rise to clonal expansion in the small intestinal crypt^9^,^10^. The mathematical analysis of clonal expansion dynamics has revealed that oncogenic mutations in Lgr5-positive cells alter the neutral drift pattern governing Lgr5-positive stem cell replacement, resulting in an elevated expansion potential for mutated cells^11^,^12^. Moreover, these models have been also adopted for the study of cell clonal expansion in colonic human epithelium based on the ribbon width of the clonal imprints derived from somatic mtDNA mutations^13^.

Proliferation of epithelial cells in the gastrointestinal tract has been assessed by counting cells in arrested metaphase, as well as counting labelled cells with thymidine analogues and/or other division labels^14^,^15^,^16^,^17^,^18^. The estimation of proliferation rates base on the rate of accumulation of cells in arrested metaphase over time is a straightforward technique^14^,^15^. The estimation of kinetic parameters by monitoring cells labelled with a label pulse during S phase^14^,^15^ can be carried out by estimating the duration of the time intervals between peaks of labelled cells generated by the division of the initially labelled cells^16^,^18^,^19^ or by fitting mathematical models specifically describing this labelling process^20^,^21^. These techniques are useful for assessing the entire proliferative compartment in a tissue, but are inherently inaccurate for studying the kinetics of cell subpopulations because of the requisite assumption that cell lineages can be identified on the basis of their position within the tissue. In addition, labelled populations can only be followed for a limited number of cell cycles because the label is diluted during each division. Using these techniques it is not possible to get information regarding cell differentiation.

In this work, we have generalized the solution of clonal expansion models to accommodate any value or distribution for the initial number of labelled cells in lineage tracing experiments and proposed the use of the limiting probabilities of clonal expansion for parameter estimation. In addition, we are introducing the use of stochastic birth-death and dynamic compartmental models to quantify cell proliferation and differentiation of Lgr5-positive cells and their progeny. This methodology enables an exhaustive quantification of the temporal dynamics of selected cell populations in the gastrointestinal epithelium, which we have applied here to quantify proliferation, differentiation and clonal expansion of Lgr5-positive cells in pyloric glands. Moreover, we have proven the performance of this model under altered situations in which stem cell proliferation and differentiation are uncoupled simulating gastric disease conditions.

## Results

### Lineage tracing analysis of Lgr5-positive cells in pyloric gastric glands

**Figure 1:**
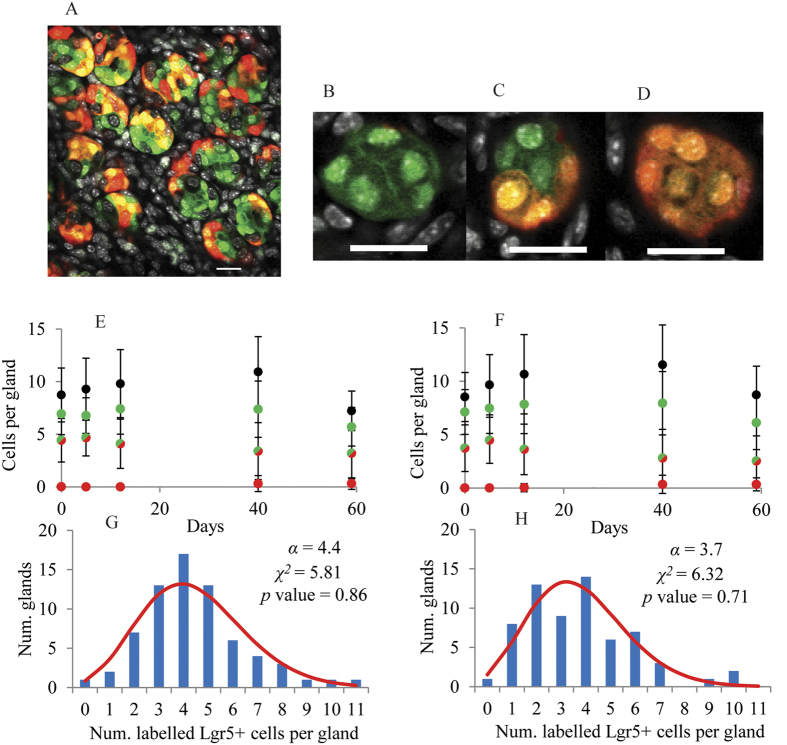
Lineage tracing of Lgr5-positive cells within pyloric gland bases. (**A**) XY plane image or bottom view of the pyloric epithelium after removing the gastric muscle layer at 42 days after Cre activation of tdTomato labelling of Lgr5-positive cells by tamoxifen; (**B**) Gland base with non-tdTomato labelled Lgr5-positive cells, (**C**) with both tdTomato labelled and non-labelled Lgr5-positive cells and (**D**) with all Lgr5-positive cells labelled with tdTomato. Scale bars represent 20 μm; Cell counts of pyloric gland bases located on the greater (**E**) and on the lesser (**F**) stomach curvature: Total cell counts (black), total number of Lgr5-positive cells (green), number of tdTomato labelled Lgr5-positive cells (green/red) and number of tdTomato labelled Lgr5-negative cells (red). Error bars represent the standard deviation of counts; (**G,H**) Distribution of the number of tdTomato labelled Lgr5-positive cells per gland immediately after Cre induction on glands located on the greater (**G**) and on the lesser (**H**) curvatures. Columns show the observed absolute number of glands for a given number of tdTomato labelled Lgr5-positive cells. Lines show the Poisson distribution with parameter *α* = 4.4 and 3.7 cells as estimated for the greater and lesser curvature, respectively. A *χ*^*2*^ test showed no significant difference (*p* value > 0.05) between the observed empirical distribution (columns) and the fitted Poisson distribution (lines) in any case.

Lgr5-eGFP-IRES-CreERT2; Rosa26-floxed-tdTomato mice were used to generate new data for the estimation of clonal expansion parameters in pyloric glands^22^. We used short-term tamoxifen exposure for induction of Cre-mediated recombination to selectively mark Lgr5-positive cells *in vivo* with an extra fluorescent protein. Accordingly, mice were treated with tamoxifen to genetically label Lgr5-positive cells with tdTomato. During a period of two months following tamoxifen induction, the pyloric epithelium was collected at several sampling times for the bottom view observation of glands located on the greater and on the lesser curvatures of the pyloric stomach using confocal microscopy (Fig. 1A–D) as previously described^3^. Cell counts of tdTomato labelled and non-labelled Lgr5-positive cells were acquired on 737 and 477 glands on the greater and lesser curvature, respectively, at day 0, 5, 12, 42 and 59 post Cre induction, (Supplementary Figure 1).

There were, on average, 9.1 ± 2.8 and 9.8 ± 3.0 cells counted in the bottom view or base of pyloric glands located on the greater (Fig. 1E) and lesser (Fig. 1F) stomach curvature, respectively. Of these, 6.9 ± 2.2 and 7.4 ± 2.7, respectively, were Lgr5-positive cells as assessed by confocal fluorescent microscopy (Fig. 1E,F). The relatively large deviation in cell counts is due to the inherent variation in size and shape of pyloric glands. There was no significant difference between cell counts on glands located on the lesser and on the greater stomach curvature. Immediately after Cre induction, 4.4 and 3.7 Lgr5-positive cells were labelled on average per gland with tdTomato on the greater (Fig. 1E,G) and lesser (Fig. 1F,H) stomach curvature, respectively.

If tamoxifen randomly induces the recombination events resulting in tdTomato labelling of Lgr5-positive cells, the number of labelled cells per gland should have a Poisson distribution. A test of goodness of fit indicated that there were no significant differences (*p* value > 0.05) between the observed empirical distribution of the initial number of Lgr5-positive cells labelled with tdTomato per gland or initial clone size and the fitted Poisson distribution on either curvature (Fig. 1G,H). These results support randomness of tamoxifen induced tdTomato labelling of Lgr5-positive cells in glands.

tdTomato labelled Lgr5-positive cells proliferate and eventually may differentiate. We assumed that losing Lgr5-associated GFP is an indicator of differentiation. Most Lgr5-negative cells are located above the gland base. However, we observed, on average, 1–3 Lgr5-negative cells at the base of the gland intermingled with Lgr5-positive cells (Fig. 1E,F). These cells were eventually replaced by descendants of tdTomato labelled Lgr5-positive cells. This cell replacement process was slow, as evidenced by the fact that only a few tdTomato labelled Lgr5-negative cells were detected at 42 and 59 days post Cre-induction (Fig. 1E,F). This is already indicative of a relatively slow cell replacement rate at the base of the glands.

### Clonal expansion of descendants of Lgr5-positive cells in pyloric glands

Lopez-Garcia (2010)^8^, confined the study of clonal expansion in the small intestinal crypt to the bottom of crypts and justifed it with the argument that clone expansion along the vertical crypt axis occurs at a rate that far exceeds clone expansion across the crypt base, and as a consequence, the rate-limiting process that governs monoclonal expansion to the entire crypt is the expansion in the base of the crypt. In the stomach, Lgr5-positive cells located at the base of pyloric glands and their descendants are able to occupy the entire gland^2^. Previous work has demonstrated much faster expansion of clones along the vertical crypt axis than at the basal plane^3^. Hence, the reasoning previously applied to intestinal crypts can be applied to Lgr5-positive cells located at the base of pyloric glands. The successful clone will have to occupy the base of the gland, which is a much slower process than the vertical expansion of the clone. Based on these observations, we conclude that the model developed by Lopez-Garcia (2010)^8^ to study clonal expansion in small intestinal crypts is applicable to study the expansion of Lgr5-positive cell descendants in pyloric glands, and data collection and analysis can be limited to the pyloric gland base region or “bottom view” as previously described^3^.

The model of Lopez-Garcia (2010)^8^ hypothesises that equipotent clones arise from *N* Lgr5-positive cells located at the base of the gland and evolve until either they are extinguished or they expand to the entire gland. As demonstrated in the intestine^11^,^23^, the number of Lgr5-positive cells able to give rise to monoclonal crypts does not have to be the same as the total number of Lgr5-positive cells in crypts or glands. This model hypothesizes that the rate at which clones gain cells is equal to the rate at which they lose cells, *λ*. This rate can be also interpreted as the clone replacement rate because the gain or loss of cells in one clone is balanced with the loss or gain of cells in other clones. Gaining and losing clone cells is associated with cell proliferation but as discussed below these are two processes with different dynamics.

#### Modelling clonal expansion with initial clone sizes greater than one cell

In previous studies, monoclonal expansion of small intestinal stem cells has been modelled in situations in which the initial number of cells per clone was always equal to 1 cell^8^,^10^,^11^,^12^,^23^. The initial clone size can be a fairly constant number equal to 1 in *in vivo* experiments activated with relatively low doses of tamoxifen that ensure a very low number of recombination events. However, at higher doses, the initial number of cells will be higher than 1 cell and may vary from clone to clone. The application of the clonal expansion model previously reported^8^ to fit datasets that have initial clone sizes greater than 1 cell is possible under the assumption that all those cells per gland simultaneously labelled after Cre induction behave as a clone. This assumption is plausible because one of the model hypotheses is that the initial *N* cells at the base of the gland are equipotent and have the same behaviour regarding expansion and independently of their predecessors. If this is true for all *N* cells at any time, there should not be any difference in the expansion behaviour of a group of Lgr5-positive cells simultaneously labelled after Cre induction and the behaviour of a group of clonal cells derived from the same labelled predecessor. Initially labelled cells post Cre induction will not typically be in contiguous positions; but again according to the hypothesis that cells are equipotent, contiguously labelled cells should still follow the same expansion dynamics as mosaic-labelled or discontinuous clones.

**Figure 2:**
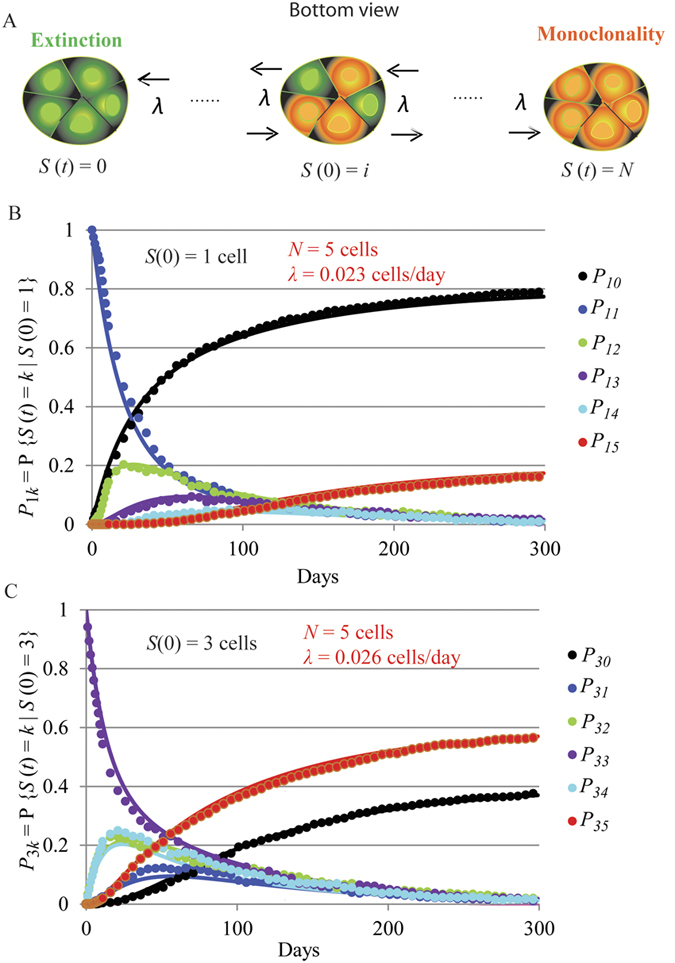
Modelling clonal expansion dynamics when the initial size of the clone is greater than 1 cell. (**A**) The model hypothesises that equipotent clones rise from *N* Lgr5-positive cells located at the base of the gland. The initial clone size, *S*(0) can take any number from 1 to *N*. Clone size changes at a rate *λ* until reaching extinction, *S*(*t*) = 0, or monoclonality, *S*(*t*) = *N*. (**B**) Generated frequencies with an IBM (dots) and predicted probabilities by Equation (S5) (lines) of the clone size over time starting with 1 initial cell per clone and (**C**) with 3 initial cells per clone randomly located at the base of the gland. The value of the parameters used for data generation in the IBM model were 0.02 cells/day for the clone replacement rate, *λ*, and 5 cells for the number of clone-producing cells at the base of the gland, *N*. The values of the parameters recovered by fitting the model in equation (S5) to these datasets are shown in red on the plots. Clones were monitored in one thousand glands simulated during 300 days for each initial clone size.

We have generalized the explicit solution of the previously reported model of clonal expansion^8^ for cases in which the initial number of Lgr5-positive cells labelled per clone was greater than 1 (see supplementary material). As shown in Fig. 2A and in Equations (S5), derived in supplementary material, with this generalization the process of clonal expansion can be described by the parameters *λ* and *N* plus a third parameter, the initial clone size, *i*. Furthermore, it is necessary to consider that the initial number of cells exhibiting the recombination derived label varies from clone to clone, i.e. the initial clone size will have a distribution of values. Suitable distribution functions such as the Poisson and binomial distributions as well as the empirical distribution of the sample can be estimated from the data and used as - initial state. The resulting equations to describe the expansion of clones over time in pyloric glands when the initial clone size distribution is equal to *F* (*i*) are:


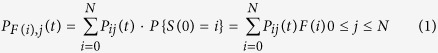


where *P*_*ij*_ (*t*) = *P*{*S*(*t*) = *j* |*S*(0) = *i*}, expresses the probability that the clone size, *S*, is equal to *j*, which takes values between 0 and *N*, at time *t > *0 given that the initial clone size, *S*(0), is equal to *i* = {0 .. *N*}. The detailed mathematical expressions are in Equations (S8) in the Supplementary material.

In agreement with this, the results of fitting Equations S5 to clonal expansion data generated with an individual based model (IBM) developed for the pyloric gland showed that the estimation of clonal expansion parameters is not affected by the distribution pattern of the initially Cre-labelled Lgr5-positive cells. The estimated replacement rate and the number of cells giving rise to monoclonality used for data generation with the IBM were very similar when fitting both i) datasets generated with glands in which one randomly chosen Lgr5-positive cell per gland was labelled (Fig. 2B) and ii) datasets generated with glands in which 3 Lgr5-positive cells per gland were randomly labelled and thus not contiguously located in most of the cases (Fig. 2C). A thousand glands were simulated for each scenario by the IBM with identical parameters.

Supplementary material and Supplementary Figure 2A–D show the importance of using the exact initial number of labelled cells post Cre induction per clone to fit clonal expansion models. Assuming initial clone sizes that are different from the actual initial number of cells driving the process of clonal expansion will considerably increase the error of estimation of parameters *N* and *λ*.

#### Estimation of the number of Lgr5-positive cells at the base of the gland able to give rise to monoclonality, N, by fitting the probability of clone extinction to long term experimental data

The probability of clone extinction can be estimated from the limiting behaviour of Equations (S5) if the initial clone size is constant or Equations (S8) if it varies according to a distribution function, respectively (see supplementary material). The resulting limiting probability functions can be used to estimate the number of Lgr5-positive cells at the base of the gland able to give rise to monoclonal glands, *N*. As demonstrated in the supplementary material, the probability of clone extinction depends on the number of Lgr5-positive cells at the base of the gland able to expand, *N*, and on the initial clone size, *i*, but it is not affected by the clone replacement rate, *λ*, and it is expressed as follows:


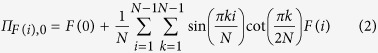


where 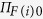
is the probability of clone extinction in glands given that the initial clone size has a distribution *F*. The derivation of this expression can be found in Supplementary Material.

**Figure 3:**
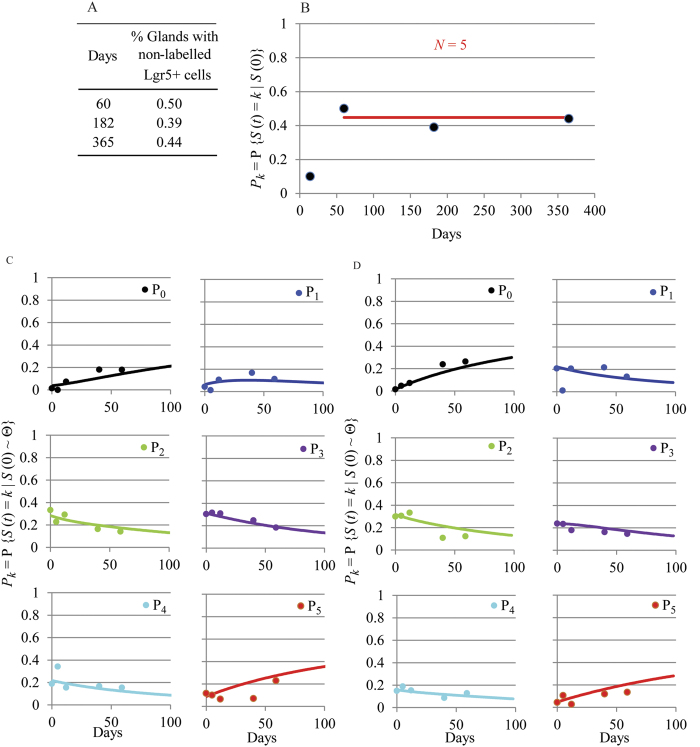
Estimation of clonal expansion parameters. (**A**) Dataset from a long term lineage tracing 4 colours experiment after tamoxifen induction and previously reported 3 used to estimate the number of Lgr5-positive cells, *N*, giving rise to clonal expansion in pyloric glands by fitting the probability of extinction. The table shows the percentage of glands with non-Cre labelled Lgr5 + cells over the total number of glands analysed in the last three sampling times when limiting behaviour was observed. (**B**) The red line shows the predicted probability of clone extinction, Π_*PP(α)0*,_ fitted to the data points (dots) reported in (**A**). The initial value for the clone size was a Poisson distribution with parameter *α* = 2.3 cells. The fitted value of *N* was equal to 5. (**C,D**) Estimation of clone replacement rate, *λ*, at the base of pyloric glands by fitting the probability distribution functions (lines) to the observed relative frequencies of the number of tdTomato labelled Lgr5-positive cells (dots) per gland base over time on the greater (**C**) and on the lesser (**D**) stomach curvature newly generated in this work and reported in Supplementary Figure 1; *P*_Θ(*i*) *k*_ = P {*S* (*t*) = *k*|*S* (0) ~ Θ(*i*)} is the probability that the number of labelled Lgr5-positive cells at time *t*, *S* (*t*) is equal to *k*, given that *S* (0) has the empirical distribution estimated form the data at day 0 after tamoxifen induction Θ(*i*). The value of *N* was fixed to 5 as previously estimated (Fig. 3B).

We fitted Equation (2) to a previously published dataset derived from the bottom view confocal microscopic observation of pyloric glands in a long term 4 colours lineage tracing experiment^3^ using adult Lgr5-eGFP-IRES-CreERT2; Rosa26-floxed-four colour animals^23^. Figure 3A shows the dataset used to fit this probability function. This dataset includes the percentage of Lgr5-positive glands with non-Cre labelled cells observed at all sampling times. Therefore, clone extinction was quantified from the proportion of glands in which clones were displaced from the base of the gland and become extinct over time. Importantly, the experimental period in which this dataset was collected was long enough for the process to exhibit limiting behaviour (Fig. 3B).

An accurate estimation of the initial clone size is essential for the estimation of the parameter *N* by fitting Equation (2) (see Supplementary material and Supplementary Figure 2A–E). Figure 1G,H show good agreement between the distribution of the initial number of Cre-labelled cells per gland and the Poisson distribution. Thus, we assumed that the recombination events that result in the initial labelling of Lgr5-positive cells with one of the 4 colours were a set of 4 independent superposed Poisson processes. It can be demonstrated that the sum of independent superposed Poisson distributed variables has also a Poisson distribution with parameter equal to the sum of the parameters of the original random variables. Then, the total number of Lgr5-positive cells initially labelled with any Rosa derived colour per gland was assumed to have a Poisson distribution with parameter *α*, so that in Equation (2) *F* (*i*) = 
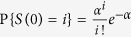
. The value of *α* was estimated from the initial percentage of glands with non-Cre labelled Lgr5-positive cells, i.e. *α* = 2.3 cells. In that experiment, measurements were not recorded immediately after tamoxifen activation but 12 days post Cre induction. Based on the relatively slow process of clonal expansion observed in the newly generated datasets, we disregarded the error associated with possible extinction within the first 12 days post Cre induction. Under these premises, the resulting estimation for *N* was 5 ± 0.19 cells (Fig. 3B). The analysis of the MCMC fitting results can be found in Supplementary Figure 3.

#### Estimation of clone replacement rate at the base of the gland, λ, in the pyloric gland under the hypothesis of neutral-drift dynamics

The newly generated data in this work shown in Fig. 1 and Supplementary Figure 1 was collected for a period of 2 months after tamoxifen administration in Lgr5-eGFP-IRES-CreERT2; Rosa26-floxed-tdTomato mice^22^. This period of time was not long enough for pyloric glands to exhibit limiting behaviour i.e. monoclonality. For instance, two months after tamoxifen injection, a large number of glands had similar proportions of both labelled and unlabelled Lgr5-positive cells so that clonal expansion of Cre-labelled cells had not yet been resolved (Supplementary Figure 1). The simultaneous estimation of clonal expansion parameters, *N* and *λ*, is not straightforward with this dataset. As *N* was already estimated from the probability of extinction as described above, the measurements from the transient period of the process were used to estimate *λ*.

The newly generated dataset in this work comprises counts on labelled glands located on the greater and lesser curvature of the pylorus. We decided to fit these two datasets separately although no great differences were expected since the location of glands did not seem to affect the number or labelling of Lgr5-positive cells (Fig. 1). To fit Equations (S8), *N* was fixed to 5 cells as previously estimated (Fig. 3B) and the empirical distribution of the initial clone size was estimated from the data. The fitting procedure is described in detailed in Supplementary Material and the analysis of the MCMC fitting results can be found in supplementary Figure 4A,B. Figure 3C,D show good agreement between the model in Equations (S8) and the experimental data gathered on the greater and lesser curvature of the pyloric region, respectively. Thus, provided that a one dimensional-random walk with boundaries is a good approximation to model clone size, these 5 cells are equipotent in the sense that they have the same probability of being replaced by an expanding neighbour as derived from the neutral drift model hypothesis.

The estimates for the clone replacement rate at the base of pyloric glands were 0.017–95% credible interval, CI, is (0.014–0.02) -and 0.020 -CI is (0.016–0.025)- cells per day in glands located within the greater and lesser pyloric stomach curvatures, respectively. Considering the experimental error, these rates did not differ significantly from each other (supplementary Figure 4C). Thus, clones at the base of pyloric glands on average, gain or lose one cell approximately every 50–58 days.

### Proliferation and differentiation of cells within pyloric gastric glands

Using clonal expansion analysis we have identified 5 effective Lgr5-positive cells at the base able to expand to the entire gland; clones involving these 5 cells lose and gain 0.02 cells per day by random replacement. Cell replacement of these clone-producing cells is coupled with cell proliferation but these are two different processes; one involves cell production and the other cell displacement in a confined geometry. Not all proliferation events affecting clone-producing cells necessarily involve the replacement of one of them as already demonstrated in the intestinal crypt, where a clone gains/loses a cell every 5–6 days^10^ while stem cells divide once a day^18^. The rates governing these processes are also different; the rate at which clones expand and shrink at the base of the gland describes the number of cells that the clone gains/loses per unit time, and this rate is independent of the size of the clone. Symmetric cell division implies exponential growth with a rate that depends on the number of proliferative individuals in the population. The specific growth rate is defined as the number of newly generated cells per cell within the population in a unit of time.

For the analysis of clonal expansion, Cre-labelled Lgr5-positive cells located in the base of the gland need to be quantified; all glands within the bottom view image need to be considered, including those with no Cre-labelled cells which are essential to track clone extinction over time. For cell proliferation and differentiation studies, the total number of Lgr5-positive and Lgr5-negative Cre-labelled cells per gland is required but only from Cre-labelled glands. In addition, models comprising accurate hypotheses for cell proliferation and differentiation have to be applied. We used a birth-death stochastic model^24^ to describe proliferation and death or differentiation of the Lgr5-positive population. Cell proliferation was assumed to be driven by symmetric cell division, which is in agreement with previous results in glands where 90% of Lgr5-positive cells were reported to undergo symmetrical division^3^. Cell differentiation was identified by the loss of the GFP associated with Lgr5 expression. We assumed that differentiated cells do not reacquire this marker. Cell reacquisition of Lgr5 has been described in small intestinal crypts after deletion of Lgr5-positive stem cells^25^,^26^ implying a high level of plasticity of the stem cell progeny. No specific information is available for the stomach; however in a healthy gut in homeostasis, reacquisition of Lgr5 after cell differentiation is unlikely to be a frequent event.

As detailed in the supplementary material, the solution of the stochastic differential equations of the birth-death process when the initial number of labelled Lgr5-positive cells per gland is equal to 1 and the proliferation rate, *μ*, is different from the differentiation rate, *δ*, is as follows^24^:


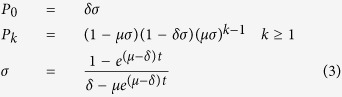


With *P*_*k*_ = P{*L*^*+*^(*t*) = *k*|*L*^*+*^(0) = 1} where *L*^*+*^(*t*) is the number of labelled Lgr5-positive cells at time *t* and *L*^*+*^(0) is the number of labelled Lgr5-positive cells immediately after Cre induction. The solution of the stochastic differential equations of the birth-death process is different when *μ = δ* (see supplementary material)^24^. In this case Equations (3) have to be replaced by


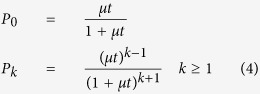


**Figure 4:**
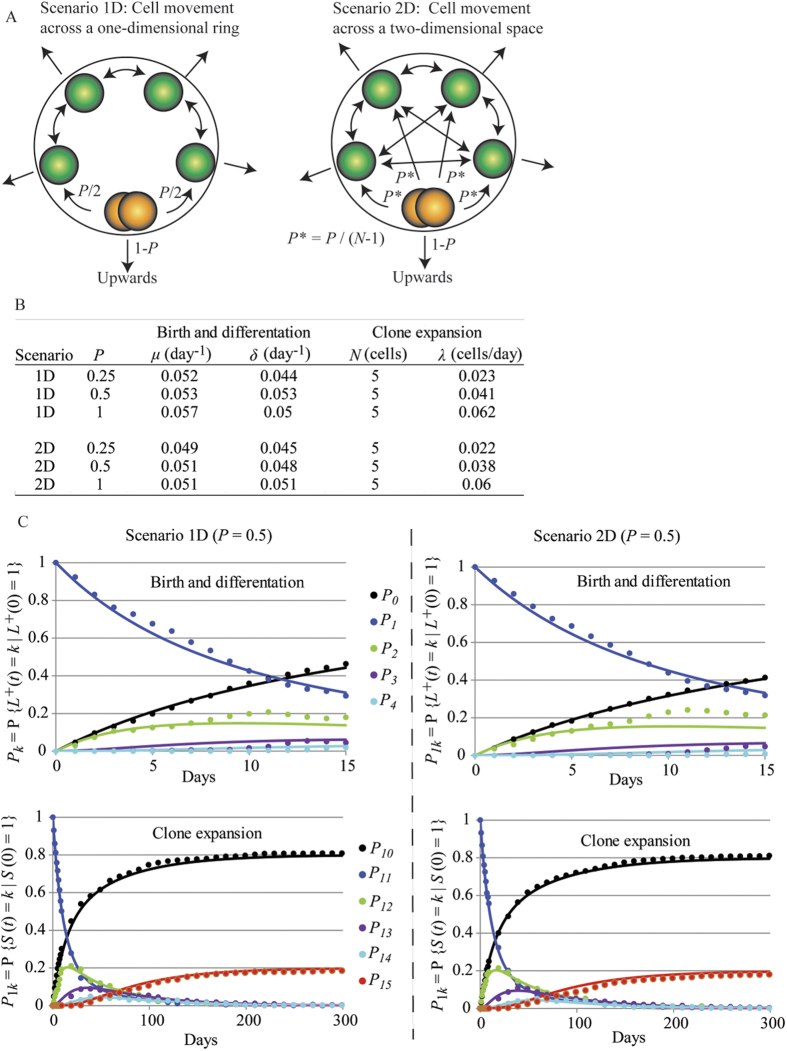
Simulating cell dynamics at the base of the gland. (**A**) Diagram of two possible scenarios for cell replacement of Lgr5-positive cells at the base of the gland implemented in the IBM. After cell division, cell replacement of effective Lgr5-positive cells takes place with probability *P* in either a one dimensional ring (scenario 1D) or in two dimensions (scenario 2D); otherwise the newly generated cell moves upwards with probability 1-*P*. (**B**) The table shows the value of the parameters of the birth-death model in Equations (3) and the clone expansion model in Equations (S5) estimated by fitting datasets generated in both scenarios, with several values for the probability of cell replacement at the base of the gland. The initial number of labelled cells or clone size was 1 cell; the values of all other parameters of the IBM were equal for all simulations. (**C**) Fitted models (lines) for proliferation-differentiation and clonal expansion of Lgr5-postive cells in pyloric glands to data (dots) simulated with the IBM assuming that after division Lgr5-positive cells located in clone producing positions replace each other with probability *P* = 0.5 and the spatial reorganization takes place in either one (Scenario 1D) or two dimensions (Scenario 2D). One thousand glands were simulated for 300 days for each set of conditions. Models were fitted to data by MCMC methods.

To further explore cell dynamics, simulations were run with the IBM implemented for pyloric glands (see supplementary material). The IBM assumes that there are 7 Lgr5-positive cells proliferating and differentiating at the base of the gland. Of them, the 5 Lgr5-positive cells located at the basal position are considered to be the effective clone-producing cells. The division cycle of Lgr5-positive cells was assumed to have a gamma distribution with an average duration of 11 days, thus the specific growth rate of the population is approximately 0.06 per day^27^. After division, clone-producing cells can replace each other with probability *P* or the newly generated cell can move to the ring immediately above with probability 1-*P*. Several values for *P* were assayed in two scenarios for the spatial replacement of clone-producing cells (Fig. 4A). In the first scenario i) cell replacement affects either the cell on the left or on the right with the same probability, i.e cells move along a one-dimensional ring; in scenario ii) cell replacement takes place in two dimensions, so that any of the other effective Lgr5-positive cells have the same chance to be replaced. In both scenarios, the just replaced cell could either move upwards with probability 1-*P* or could replace one of the other effective Lgr5-positive cells. For instance, under these premises, if *P* = 1, the two newly generated cells remain located in the 5 basal clone producing positions and the cell displacement spreads across the entire base so that all clone-producing cells are relocated; when *P* = 0.5, the chance that both newly generated cells remain in clone-producing positions is 50% and relocation will involve 1–2 cells only; if *P* = 0.25, one of the newly generated cells moves upwards in 75% of the cases and when cell replacement affects basal cells the spreading of cell displacement is very limited. Figure 4B shows that slightly smaller values for the specific birth and death rates were observed for small values of *P*, but in general the estimation of cell proliferation and differentiation parameters was not affected by the simulation conditions. Regarding clonal expansion parameters, the rate at which clones gain and lose cells was strongly influenced by the value of *P*, while the spatial re-organization in one or two dimensions did not have any effect (Fig. 4B,C). When *P* = 1, the numerical value of the rate of clone replacement, *λ*, was similar to that of the specific growth rate of the population, *μ*; while this numerical coincidence is expected, situations in which *P* = 1, i.e newly generated clone-producing cells replace exclusively other clone-producing cells, would not be expected *a priori* in homeostatic conditions.

#### Quantifying proliferation and differentiation of Lgr5-positive cells in pyloric glands

**Figure 5:**
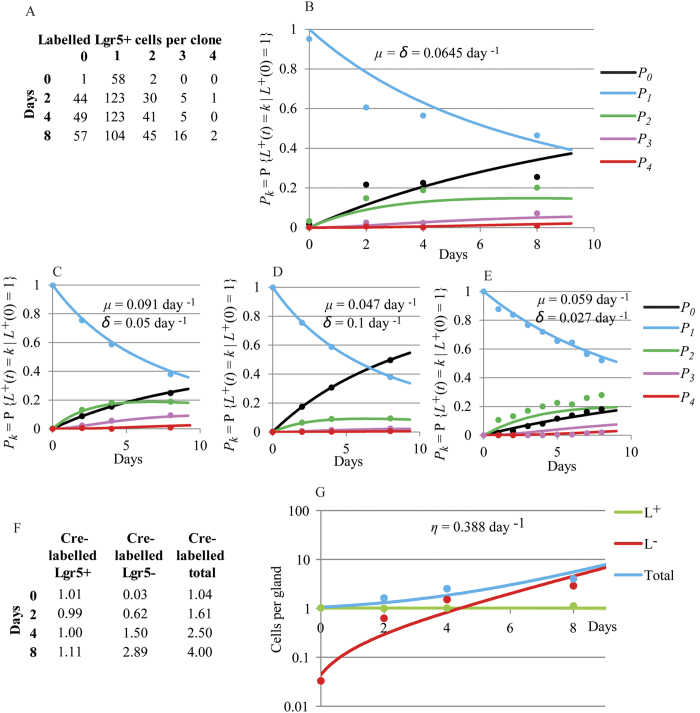
Estimation of proliferation and differentiation rates of Lgr5-positive cells and their progeny in pyloric glands. (**A**) Dataset generated from a short term lineage tracing 4 colours experiment previously reported^3^. The matrix shows the absolute number of clones with a given number of Lgr5-positive cells labelled with the same Cre-induced colour over time; (**B**) Fitted probability distribution functions (lines) (Eq. 4) to the observed relative frequencies of the number of labelled Lgr5-positive cells (dots) per gland with the same Cre-induced colour over time reported in A); *P*_*k*_ = P{*L*^*+*^(*t*) = *k*|*L*^*+*^(0) = 1} where *L*^*+*^(*t*) is the number of Cre-labelled Lgr5-positive cells at time *t* and *L*^*+*^(0) is their initial number. The fitted model assumes that the proliferation rate of Lgr5-positive cells, *μ*, is equal to their differentiation rate, *δ*. (**C,D**) Fitted probability distribution functions (lines) to predicted relative frequencies of Cre-labelled Lgr5-positive cells (dots) in pyloric glands using Equations (3) with *μ* = 0.1 day^−1^ and *δ* = 0.05 day^−1^ (**C**) and *μ* = 0.05 day^−1^ and *δ* = 0.1 day^−1^ (**D**). Fitted values for *μ* and *δ*, shown on the plots, were similar to the true values used to generate data; (**E**) Fitted probability distribution functions (lines) to simulated frequencies of Cre-labelled Lgr5-positive cells (dots) in pyloric glands with the IBM assuming 7 initial Lgr5-positive cells located in the gland base with *μ* = 0.063 day^−1^; simulated differentiation and proliferation were uncoupled with slower differentiation than proliferation rates. Fitted value for *μ* and *δ* are shown on the plot. One thousand glands were simulated. (**F**) Dataset generated from the same experiment as dataset in (**A**,**B**). The matrix shows the average number of Lgr5-positive and Lgr5-negative cells labelled with the same Cre-induced colour per gland over time; (**G**) The compartmental model (Eq. 5) was fitted (lines) to observations in (**F**) (dots) on the number of Lgr5-positive (green), Lgr5-negative (red) and total (blue) cells labelled with the same Cre-induced colour per gland. The values of *μ* and *δ* were fixed to that previously obtained in (**B**). Models were fitted to data by MCMC methods.

A suitable dataset to study proliferation and differentiation of Lgr5-positive cells is shown in Fig. 5A. This dataset has been derived from the short term 4 colour lineage tracing experiment previously reported^3^. In those experiments, adult Lgr5-eGFP-IRES-CreERT2; Rosa26-floxed-four colour animals^23^ were administered with tamoxifen to activate one of the four different fluorescent reporter genes at random; consequently individual Lgr5-positive cells acquired one of the 4 colours as a permanent heritable fluorescent label. Dataset in Fig. 5A describes the frequency of having a given number of Lgr5-positive cells labelled with the same colour per gland over time. The applied dose of tamoxifen aimed to obtain a maximum number of glands with one unique Lgr5-positive cell labelled with one of the four Rosa-derived colours. Indeed, the dataset in Fig. 5A shows that at day 0, we detected only one Lgr5-positive cell labelled with one of the four Rosa-derived colours in practically all glands showing recombination events which fully met the initial value requirement of the model in Equations (3).

Supplementary Figure 5A shows the analysis of the Monte Carlo Markov Chain (MCMC) results of fitting Equations (3) to dataset in Fig. 5A and a statistical summary for the estimates of the proliferation and differentiation rate as well as for the difference between both. The values for the proliferation and differentiation rate were 0.068 day^−1^ −95% credible interval is (0.058–0.079)- and 0.061 day^−1^ (0.051–0.071), respectively. The difference between the rate of proliferation and the rate of cell differentiation was not significant (Supplementary Figure 5A) and Equations (4) were fitted to estimate the exact value of the specific growth -differentiation- rate of the population which was equal to 0.064 (0.057–0.073) day^−1^ (Fig. 5B, Supplementary Figure 5B). Therefore, the doubling time of the Lgr5-positive population in pyloric glands is approximately 10.7 days, which is also the time for a cell to differentiate on average. The equilibrium between cell proliferation and differentiation is expected in a healthy gut in homeostasis where cell populations are in steady state and maintain constant numbers.

In order to test the model performance in altered situations in which the Lgr5-positive population is not in steady state, predicted relative frequencies of Lgr5-positive cells in pyloric glands with *μ* = 0.1 day^−1^ and *δ* = 0.05 day^−1^ (Fig. 5C) as well as with *μ* = 0.05 day^−1^ and *δ* = 0.1 day^−1^ (Fig. 5D) were obtained using the model in Equations (3). By fitting the model to the generated frequencies, we observed that the estimations of the parameters *μ* and *δ*, were very similar to the true values used to generate the data (Fig. 5C,D). Moreover, the IBM developed for the pyloric gland (see Supplementary material) was used to simulate the dynamics of the Lgr5-positive population, assuming 7 initial Lgr5-positive cells residing at gland basal positions with cell cycle duration equal to 11 days, which results in a population proliferation rate, *μ*, equal to 0.063 day^−1^. During simulations, newly generated Lgr5-positive cells did not differentiate while located in the first 14 positions of the gland base. This condition was sufficient to uncouple Lgr5-positive cell differentiation and cell proliferation, resulting in slower rate of differentiation. By fitting the model in Equations (3) to the simulated frequencies, we observed that the estimated value for *μ* was similar to the true value used to generate the data, while the fitted value for *δ* was approximately half the value of *μ* (Fig. 5E). These results demonstrate the application of the model for detecting loss of homeostasis in the Lgr5-positive cell population in mouse gastric disease models.

#### Quantifying proliferation of Lgr5-negative cells in pyloric glands

To complete the quantification of the temporal cell dynamics in pyloric glands, a compartmental model was developed to estimate the specific proliferation rate of the population of the descendants of Lgr5-positive cells after differentiating or losing Lgr5, i.e. Lgr5-negative cells exhibiting a Cre-induced label. As above, we have assumed that differentiated cells do not reacquire this marker. The number of Lgr5-positive, *L*^*+*^, and Lgr5-negative, *L*^−^, cells labelled with the same Cre-derived colour per gland over time was modelled as follows:


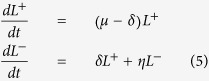


where *η* is the specific growth rate of the differentiated cell population and the other parameters have been defined above.

Equations (5) can be solved explicitly for given initial values as detailed in the supplementary material. The value of *η* was estimated by fitting the solution of Equations (5) to a dataset with the observed total cell counts of both Lgr5-positive and Lgr5-negative cells labelled with the same colour per gland at several times shown in Fig. 5F. This dataset was generated from the same previously published experiment^3^ as dataset in Fig. 5A. The dataset used to fit Equations (5), needs to be derived from the experiment used to estimate the proliferation and differentiation rate of Lgr5-positive cells. This is because the number of Lgr5-negative cells depends on their own proliferation as well as on the differentiation of Lgr5-positive cells. Moreover, the sampling period has to be restricted so that Cre-labelled cells are not lost by migration out of the pyloric glands.

In agreement with the balance between cell proliferation and differentiation reported above for a healthy gut in homeostasis, the dataset in Fig. 5F,G shows that the mean number of Lgr5-positive cells labelled with the same colour per gland maintained its value, equal to 1 cell in this case, fairly constantly over time. This was also observed in the lineage tracing dataset newly generated in this work using a different reporter mouse model (Fig. 1E,F).

The solution of Equations (5) was fitted to dataset in Fig. 5F by fixing the value of *δ* and *μ* to 0.064 day^−1^ which was the value estimated for the differentiation and the proliferation rate of Lgr5-positive cells from this experiment (Fig. 5B). The analysis of the fitting results is shown in supplementary Figure 6. The estimated specific growth rate for Lgr5-negative cells was *η* = 0.39 day^−1^ −95% CI is (0.35–42)- (Fig. 5G). Thus, the time required for a population of Lgr5-negative cells to double its number is approximately 1.8 days.

## Discussion

The solution of the model for clonal expansion^8^ of initial clones with size greater than 1 is important when working with animal models in which it is difficult to know *a priori* the efficiency of the genetic recombination process leading to the labelling of cells in a pyloric or intestinal unit. This solution is based on the assumption that a group of Lgr5-positive cells simultaneously labelled post Cre induction behave as a clone. These cells reflect independent induction events and it could be argued that in a strict sense at the time of labelling induction they are not clonal; however tracing experiments have proven that monoclonal expansion of Lgr5-postive stem cells is a steady state process^2^,^8^. This means that a common clonal ancestry could be always identified for all cells within a gland/crypt at any time, which implies that glands/crypts are always monoclonal in the healthy gut with respect to a past ancestor; at the same time one of the cells observed at a given time is the common progenitor for all the cells found within the gland/crypt in the future. Thus, cells simultaneously labelled after Cre induction are actually cells derived from the same ancestor.

As previously highlighted, the estimation of the individual values of the clonal expansion model parameters *N* and *λ* is not straightforward and eventually not achievable due to the resolution of the data^8^,^11^. In this work, we have shown that the *N* parameter can be independently estimated by fitting the probability of clone extinction, which does not depend on the rate of clone replacement, *λ*. Fitting limiting probabilities of clone expansion requires datasets from long term experiments showing limiting behaviour, but it results in a robust estimation of *N*, which overcomes the complications derived from the relationship between this parameter and the rate of replacement.

The reporter mouse models used in this work make it possible to quantify both Cre- labelled and unlabelled Lgr5-positive cells as well as Cre labelled Lgr5-negative cells over time. This results in an accurate estimation of the initial clone size and the frequency of clone extinction, essential prerequisites for the quantification of clonal expansion dynamics and of the differentiation of Lgr5-positive cells and the proliferation of their descendants. These estimations cannot be carried out in mouse models in which unlabelled cells resulting from lack of recombination cannot be distinguished from those cells lacking the target promoter.

Clonal expansion and cell proliferation are different dynamic processes and required different model hypotheses. We have identified a suitable mathematical model to quantify proliferation and differentiation of specific cell populations. We used this approach to estimate the proliferation rate of Lgr5-positive cells within pyloric glands as well as their differentiation rate, and concluded that they were not significantly different. This is expected for a population in steady state during homeostasis. This model could inform on the balance between proliferation and differentiation of Lgr5-positive cells in response to perturbations or disease. Regarding the relationship between cell proliferation and clone expansion, the proliferation rate of the Lgr5-positive population in pyloric glands was significantly faster than the rate at which clones expanded. This suggests that the majority of Lgr5-positive stem cell divisions serve to renew epithelial cells and only a few result in the effective replacement of a neighbour to effect expansion to the entire gland. Similar results have been reported in intestinal crypts and adenomas^10^.

The methodology proposed here effectively overcomes the limitations of previous techniques used to estimate proliferation of epithelial cells in the gastrointestinal tract^14^,^15^,^16^,^17^,^18^,^19^,^20^,^21^ and allows kinetic parameters of specific cell populations within the proliferative compartment to be estimated based on the permanent genetic labelling of the progeny.

The integration of mathematical models and cell lineage tracing experiments enables the precise quantification of population kinetics and clonal expansion dynamics of specific cell populations in the gastrointestinal epithelium in either homeostasis or altered conditions, as demonstrated for Lgr5-positive cells in the pyloric epithelium.

## Material and Methods

### Mice

Lgr5-eGFP-IRES-CreERT2; Rosa26-floxed-tdTomato mice were bred as previously described^22^. All animal experiments were approved and performed in accordance with the guidelines and regulations of the Institutional Animal Care and Use Committee of Singapore.

### Tamoxifen administration

A single dose of 0.015 mg tamoxifen/g body weight in sunflower oil (10 mg ml−1) was injected intraperitoneally in mice aged 6–8 weeks.

In all lineage-tracing experiments, experimental time zero was set to the Cre induction time, 48 h after tamoxifen injection.

### Tissue preparation and Confocal Microscopy

Samples of the lesser and greater part of the pyloric epithelium were identified anatomically as described in Supplementary Figure 7.

“xy plane” side-view imaging was performed using semithick sections of near-native tissue, generated as described in^3^.

“xz plane” bottom-view imaging was performed after removal of the muscle layer, and the epithelial tissue was directly mounted upside down in Hydromount containing Hoechst dye nuclear counterstain.

Images were acquired using an Olympus FV1000 upright confocal microscope.

### Model development

All approaches are described in detail in Supplementary Material.

### Estimation of model parameters

Parameters were estimated by finding a convergent solution of the corresponding Bayesian model using the Markov chain Monte Carlo (MCMC) random walk Metropolis algorithm implemented in SAS 9.3 software. Specific details of each Bayesian model are described in Supplementary Material.

## Additional Information

**How to cite this article**: Leushacke, M. *et al*. Quantifying Lgr5-positive stem cell behaviour in the pyloric epithelium. *Sci. Rep*. **6**, 21923; doi: 10.1038/srep21923 (2016).

## Supplementary Material

Supplementary Information
