# Supplementary material for: Quantifying Lgr5-positive stem cell behaviour in the pyloric epithelium
Source: Sci Rep. 2016 Feb 26;6:21923. doi: 10.1038/srep21923 (PMC4768140; doi:10.1038/srep21923)
Supplement: Supplementary Information [file srep21923-s1.pdf]

Supplementary Material

Quantifying Lgr5-positive stem cell behaviour in the pyloric epithelium.

Marc Leushacke[1], Nick Barker[1,2,3, §] and Carmen Pin[4, §,*]

[1]A*STAR Institute of Medical Biology, Singapore

[2]Centre for Regenerative Medicine, University of Edinburgh, Edinburgh, UK

[3]Department of Biochemistry, Yong Loo Lin School of Medicine, National University of Singapore, Singapore

[4]Gut Health and Food Safety Programme. Institute of Food Research. Norwich. UK.

Theory

1. Evolution of the clone size distribution over time (clonal expansion dynamics)

1.1. Generalizing clonal expansion models for any initial clone size

Lopez-Garcia (2010)[1], described clonal expansion in the small intestinal crypt as a stochastic process where the probabilistic dynamics of the size of the clone is that of a one-dimensional random walk with closed boundaries. This model hypothesises that the size of the clone moves at a constant rate, λ, with steps of size 1 between 0 and N cells, both of which are absorbent states [1-3]

The forward stochastic differential equations for a one-dimensional random walk with absorbent boundary states are as follows:

$$\frac{dP_{i0}(t)}{dt} = \lambda P_{i1}(t)$$
$$\frac{dP_{ij}(t)}{dt} = \lambda P_{i\,j-1}(t) - 2\lambda P_{ij}(t) + \lambda P_{i\,j+1}(\mathrm{t}) \qquad 0 < j < N \tag{S1}$$
$$\frac{dP_{iN}(t)}{dt} = \lambda P_{i\,N-1}(t)$$

Where $P_{ij}(t) = \mathrm{P}\{S(t) = j \mid S(0) = i\}$ is the probability that the clone size at time t, $S(t)$, is equal to j given that its initial size at time 0 was equal to i, $0 < i < N$. N is the number of cells in the ring and λ is the rate at which clones gain or lose cells. Therefore clonal study involves only those N cells in the gland base able to give rise to clonal expansion. By hypothesis, the clones are equipotent and follow a neutral drift dynamics so that the probability of gaining a cell is equal for all of them and equal to the probability of losing a cell. Monoclonality occurs when a given clone reaches size N, while clone takes place when the clone size reaches the other absorbent state, i.e. 0 cells.

Equations (S1) have been explicitly solved for the special case in which the initial size of the clone is 1 cell [1,3]. We have extended the solution for other initial states so that the model can be fitted to data in which the initial clone size is greater than 1 cell after tamoxifen activation.

To find the solution for any initial clone size, we started from the solution of Equations (S1) for $1 \leq j \leq N-1$ [3]:

$$P_{ij}(t) = \sum_{k=1}^{N-1} A_k \sin\left(\frac{\pi k j}{N}\right) e^{-4\lambda t \sin^2\left(\frac{\pi k}{2N}\right)}, \quad 1 \leq j \leq N-1, \quad 1 \leq i \leq N-1 \qquad \text{(S2)}$$

Where A_k is the constant of integration.

Considering that the size of a clone at the initial time, $S(0)$, is equal to i, which is between 1 and N-1, the initial value for the stochastic differential Equations (S1) are defined as follows:

$$\begin{aligned} P_{ij}(0) &= 1 \quad \text{if} \quad j = i \\ P_{ij}(0) &= 0 \quad \text{if} \quad j \neq i \end{aligned} \qquad \text{(S3)}$$

With this initial value, A_k in Equation (S2) has the form:

$$A_k = \frac{2}{N} \sum_{j=1}^{N-1} P_{ij}(0) \sin\left(\frac{\pi k i}{N}\right) = \frac{2}{N} \sin\left(\frac{\pi k i}{N}\right) \quad 1 \leq j \leq N-1, 1 \leq i \leq N-1 \qquad \text{(S4)}$$

By substituting the value of A_k in Equation (S2), the explicit solution for $P_{ij}(t)$, from Equations (S1) can be found. Replacing in Equations (S1) with the solution for $P_{ij}(t)$ in (S4), $P_{i0}(t)$ and $P_{iN}(t)$ are found by integration. Adding the transition probabilities of the absorbent states, 0 and N, the whole set of solution is then:

$$P_{i0}(t) = \frac{2}{N}\sum_{k=1}^{N-1}\sin\left(\frac{\pi ki}{N}\right)\frac{\cos^2\left(\frac{\pi k}{2N}\right)}{\sin\left(\frac{\pi k}{N}\right)}\left(1 - e^{-4\lambda t\sin^2\left(\frac{\pi k}{2N}\right)}\right),\ 1 \le i \le N-1,$$

$$P_{ij}(t) = \frac{2}{N}\sum_{k=1}^{N-1}\sin\left(\frac{\pi ki}{N}\right)\sin\left(\frac{\pi kj}{N}\right)e^{-4\lambda t\sin^2\left(\frac{\pi k}{2N}\right)},\ \ 1 \le j \le N-1,\ 1 \le i \le N-1,$$

$$P_{iN}(t) = \frac{2}{N}\sum_{k=1}^{N-1}(-1)^{k+1}\sin\left(\frac{\pi ki}{N}\right)\frac{\cos^2\left(\frac{\pi k}{2N}\right)}{\sin\left(\frac{\pi k}{N}\right)}\left(1 - e^{-4\lambda t\sin^2\left(\frac{\pi k}{2N}\right)}\right),\ 1 \le i \le N-1,$$

$$\begin{aligned} &P_{0j}(t) = 1 \quad \text{if } j = 0 \\ &P_{0j}(t) = 0 \quad \text{if } j > 0 \\ &P_{Nj}(t) = 1 \quad \text{if } j = N \\ &P_{Nj}(t) = 0 \quad \text{if } j < N \end{aligned} \qquad \text{(S5)}$$

Where P_{ik} (t) = $P\{\ S\ (t) = k \mid S\ (0) = i\}$, expresses the probability that the clone size, S, is equal to k, which takes values between 0 and N, at time $t > 0$ given that the initial or time $t = 0$ clone size, S (0), is equal to $i = \{0\ ..\ N\}$

When, i = 1, i.e. S (0) = 1, Equations (S5) are equal to the previously reported solution [1].

In the stomach, we have observed that after tamoxifen activation, the initial clone size may not be a constant value but vary from gland to gland. Thus, the variation in the initial number of labelled Lgr5-positive cells between clones has been included in the model by assuming that the initial clone size, S(0), has a given distribution $F(i) = P\{S(0) = i\}\quad 0\ \le\ i\ \le\ N$

The initial value for Equations (S1) is now

$$\begin{aligned} &P_{ii}(0) = F(i) \quad 0 \le i \le N \\ &P_{ij}(0) = 0 \quad \text{if } \ j \ne i \end{aligned} \qquad \text{(S6)}$$

And the value of A_k is equal to:

$$A_k = \frac{2}{N}\sum_{i=0}^{N}\sin\left(\frac{\pi ki}{N}\right)F(i) \qquad \text{(S7)}$$

With this value for the integrating constant, the solution of Equations (S1) is

$$P_{F(i),0}(t) = F(0) + \frac{1}{N}\sum_{i=1}^{N-1}\sum_{k=1}^{N-1}\sin\left(\frac{\pi k i}{N}\right)\cot\left(\frac{\pi k}{2N}\right)\left(1-e^{-4\lambda t\sin^2\left(\frac{\pi k}{2N}\right)}\right)F(i)$$

$$P_{F(i),j}(t) = \frac{2}{N}\sum_{i=1}^{N-1}\sum_{k=1}^{N-1}\sin\left(\frac{\pi k i}{N}\right)\sin\left(\frac{\pi k j}{N}\right)e^{-4\lambda t\sin^2\left(\frac{\pi k}{2N}\right)}F(i),\quad 1 \le j \le N-1 \qquad \text{(S8)}$$

$$P_{F(i),N}(t) = F(N) + \frac{1}{N}\sum_{i=1}^{N-1}\sum_{k=1}^{N-1}(-1)^{k+1}\sin\left(\frac{\pi k i}{N}\right)\cot\left(\frac{\pi k}{2N}\right)\left(1-e^{-4\lambda t\sin^2\left(\frac{\pi k}{2N}\right)}\right)F(i)$$

The same result can be obtained using the law of total probability as follows

$$P_{F(i),\mathrm{j}}(t) = P\{S(t)=0, S(0)\sim F\} =$$

$$\sum_{i=0}^{N} P\{S(t)=j \mid S(0)=i\}P\{S(0)=i\} = \sum_{i=0}^{N} P_{ij}(t)F(i)\quad 0 \le j \le N \qquad \text{(S9)}$$

where $P_{ij}(t)$ is defined in Equations (S5).

In the newly generated dataset in this work, the distribution of the initial size of the clones immediately after tamoxifen action was in good agreement with a Poisson distribution with parameter α (Figures 4G-H). Therefore $F(i)$ could be substituted in Equations (S8) by the following expression

$$F(i) = P\{S(0)=i\} = \frac{\alpha^i}{i!}\mathrm{e}^{-\alpha}\quad 0 \le i \le N \qquad \text{(S10)}$$

The maximum likelihood estimator for the parameter α of the Poisson distribution is

$$\hat{\alpha} = \ln\left(\frac{\text{Total clones of Lgr5+ cells}}{\text{Unlabelled clones of Lgr5+ cells}}\right) \qquad \text{(S11)}$$

When no unlabelled clones are detected, α can be estimated as follows:

$$\hat{\alpha} = \frac{\text{Number Rosa derived labelled Lgr5+ cells}}{\text{Number clones with Rosa derived labelled and unlabelled Lgr5+ cells}} \qquad \text{(S12)}$$

While the Poison distribution has a single parameter and an easily tractable form, it may not be always indicated as distribution of the initial clone size. With relatively high doses of tamoxifen, the average number of initially labelled cells after Cre activation may be close to the number of cells given rise to monoclonality, *N*. Assuming in this situation a Poisson distribution as initial clone size distribution will underestimate the probability of clones with size from 1 to N, while it will give high values to the probability of clones with a non-possible initial size, i.e. greater than *N*. In that case a binomial distribution with parameters *N*, number of cells able to give

rise to monoclonality and p, probability of having a cell labelled after Cre activation, could be used. This distributions are closely related so that when N/p is a large number, the binomial distribution converges to the Poison distribution with parameter $\alpha = N \cdot p$; Similarly, a superposed Poison distribution can be replaced by a multinomial distribution for mouse reporter models with more than one colour label induced with relatively high doses of Tamoxifen.

Other distribution functions as well as the empirical distribution derived from the histogram of observations could be used to define the distribution of the initial clone size. The empirical distribution of the initial clone size, S (0), was defined as:

$$\Theta(i) = \frac{\text{cardinal } (i \text{ at } t = 0)}{\text{total glands } (t = 0)} \qquad 0 \le i \le N$$

1.1.1 Parameter estimation

The parameters λ and N were estimated by fitting equations (S5) and (S8) to datasets, simulated or experimentally observed, respectively, by MCMC methods.

The chosen distribution function for the likelihood of the data was a multinomial with parameters $P_{i0}(t)..P_{iN}(t)$ which are described in Equations (S5) and $P_{F(i)0}(t)..P_{F(i)N}(t)$ in Equations (S8).

$$\mathrm{L}(\mathrm{Y} \mid N, \lambda) = \prod_{h=1}^{m} \text{Multinomial}(\mathrm{y}_0^h..\mathrm{y}_N^h; P_{i0}(t_h)..P_{iN}(t_h) \mid N, \lambda)$$

Where y_k^h, is the observed number of clones with k Lgr5-positive cells labelled at the h-th sampling time; $k = 0 .. N$, with N the number Lgr5-positive cells at the base of the gland; t_h is the time at which the h-th sample is taken; m is the number of sampling times; i is the initial clone size estimated from the data after tamoxifen activation.

The selected non-informative prior distributions for N and λ were:

π (N) ~ Uniform (2, 50)

π (λ) ~ Gamma ($g_1 = 0.001$, $g_2 = 0.001$)

where E [λ] = g_1 / g_2 and Var [λ] = g_1 / g_2^2

Prior to fitting the clonal model to the dataset generated in this work (Supplementary Figure 1), some adjustments need to be made to deal with the large variability of the size of the bases of the glands and accommodate models and

experimental measurements. A consideration to be made is that not all Lgr5-positive cells in the gland are able to expand to the entire gland. In the intestinal crypt between only 5-6 out of 15-16 Lgr5-positive cells are able to rise monoclonal crypts [4]. Then the measured number of labelled cells differs, in many cases considerably, of the parameter N in the model. The solution proposed for Vermeulen [3] to a similar scenario was to redistributed the probabilities P_{ij} into a fixed number of states of the random walk equal to 8 which was the constant number of fractions of the crypt used to monitor labelling expansion. In our case, we counted the number of labelled and unlabelled Lgr5-positive cells and differed considerably between glands, while the parameter N of the model or number of states in the random walk was known to have a value equal to 5 cells as estimated from a previously published long term experiment. Then the accommodation in this case was done by apportioning the observed counts in the base of the gland to the number of Lgr5-positive cells able to give rise to clonal expansion, 5 cells, and this also standardized the high variation of the size of the base of pyloric glands. This transformation required to calculate the empirical distribution of the initial clone size with the transformed data in order to fit the model.

1.2 Limiting distributions of clone size

The limiting distribution for clonal expansion can be derived from Equations (S5) when $t \to \infty$ as follows

$$\begin{aligned}
\Pi_{i0} &= \lim_{t\to\infty} P_{i0}(t) = \frac{1}{N}\sum_{k=1}^{N-1} \sin\left(\frac{\pi k i}{N}\right)\cot\left(\frac{\pi k}{2N}\right) \\
\Pi_{ij} &= \lim_{t\to\infty} P_{ij}(t) = 0, \quad 1 \le j \le N-1 \qquad \text{(S13)} \\
\Pi_{iN} &= \lim_{t\to\infty} P_{iN}(t) = \frac{1}{N}\sum_{k=1}^{N-1} (-1)^{k+1} \sin\left(\frac{\pi k i}{N}\right)\cot\left(\frac{\pi k}{2N}\right)
\end{aligned}$$

Where $\Pi_{i,k}$, $0 \le k \le N$, $1 \le i \le N\text{-}1$, is the probability of reaching a clone size equal to k when $t \to \infty$ given that the initial clone size was equal to i. The limiting probability of reaching a given clone size is equal to 0 for all cases with the exception of 0 and N which means that in the long term clone extinction and monoclonality are the only possible events and therefore their limiting probabilities add up to 1. These limiting probability functions depend on the number of Lgr5-positive cells at the base

of the gland, N, and on the initial clone size, i. They are not affected by the clone replacement rate, λ.

1.2.1 Parameter estimation

The limiting distribution assuming that the initial clone size varies in between glands according to a distribution, F, as described above is as follows:

$$\begin{aligned}
\Pi_{F(i),0} &= F(0) + \sum_{i=1}^{N-1} \Pi_{i0}(t) F(i) \\
\Pi_{F(i),j} &= 0 \qquad 1 \le j \le N-1 \qquad \text{(S14)} \\
\Pi_{F(i),N} &= F(N) + \sum_{i=1}^{N-1} \Pi_{iN}(t) F(i)
\end{aligned}$$

By assuming that the distribution of the initial size of the clones immediately after tamoxifen action has a Poisson distribution with parameter α, $PP(\alpha)$, Equations (S14) are as follows:

$$\begin{aligned}
\Pi_{PP(\alpha),0} &= e^{-\alpha} + \frac{2}{N} 1 \sum_{i=1}^{N-1} \sum_{k=1}^{N-1} \sin\left(\frac{\pi k i}{N}\right) \cot\left(\frac{\pi k}{2N}\right) \frac{\alpha^i}{i!} e^{-\alpha} \\
\Pi_{PP(\alpha),j} &= 0 \qquad 1 \le j \le N-1 \qquad \text{(S15)} \\
\Pi_{PP(\alpha),N} &= \frac{\alpha^N}{N!} e^{-\alpha} + \frac{1}{N} \sum_{i=1}^{N-1} \sum_{k=1}^{N-1} (-1)^{k+1} \sin\left(\frac{\pi k i}{N}\right) \cot\left(\frac{\pi k}{2N}\right) \frac{\alpha^i}{i!} e^{-\alpha}
\end{aligned}$$

As above any other suitable distribution function as well as the empirical observed distribution could be used.

The probability of extinction, $\Pi_{PP(\alpha),0}$, in Equations (S15) was fitted to long term experimental data by MCMC methods. In this case, the chosen distribution function for the likelihood of the data was a Binomial with parameters TGl, which represents the total number of observed glands, and $\Pi_{PP(\alpha),0}$ which is described in Equations (S15).

$$\mathrm{L}(\mathrm{Y} \mid N) = \prod_{h=1}^{m} \mathrm{Binomial}(y_h;\ TGl_h,\ \Pi_{PP(\alpha),0} \mid N)$$

Where y_h, is the observed number of glands with non-labelled Rosa derived colour Lgr5-positive cells and TGl_h is the observed total number of glands with Lgr5-positive cells within the *h-th* sample; $h = 0 .. m$, where m is the number of samples.

$\pi(N) \sim$ Uniform (2, 50) was the selected non-informative prior distribution for N.

1.3. Effect of the initial inoculum on the fitting results of the probability that the clone size has a value equal to k ={0..N} and on the fitting results of the probability of clone extinction.

To demonstrate the need of considering the exact initial clone size to fit the model in Equations (S5) and (S8), simulations were performed to evaluate the effect of the initial clone size on the estimation of the other clonal expansion parameters, N and λ. Four datasets were generated using Equations (S5) with 4 different initial clone sizes, from 1 to 4 cells. The values of the other model parameters for data simulation were fixed to arbitrarily chosen numbers, $N = 5$ cells and $\lambda = 0.1$ cells per day, in all cases. The model in Equations (S5) was then fitted to the 4 datasets assuming that the initial clone size was equal to 1 as well as that it was equal to the size used to generate the simulated data points. Table in Supplementary Figure 1A shows that the true value of the parameters N and λ was accurately recovered only when the initial clone size assumed in the model was coincidental with the initial size used to simulate the data. The model assuming 1 initial cell per clone resulted in accurate estimations of the parameters N and λ only when fitted to the dataset generated with an initial clone size equal to 1 cell (Supplementary Figures 1A and 1B). The greater the initial clone size used for data generation, the larger the bias of the estimates of N and λ obtained by fitting the model developed with 1 initial cell per clone (Supplementary Figures 1A and 1B). Supplementary Figure 1C shows that the dataset generated with 4 initial cells per clone was accurately described by the model that assumed that initial value, while the performance of the model developed with 1 single initial cell was very poor (Supplementary Figure 1D).

Moreover, the importance of using an accurate measurement of the initial clone size to fit the probability of extinction was also demonstrated by fitting Equation (2) to the dataset in Figure 3A but assuming different values for the initial clone size. Data in Supplementary Figure 3E shows that the initial clone size affects remarkably the value of the estimation of N. For instance, when the initial clone size was assumed to be a constant value for all clones equal to the measured average number of Rosa derived labelled Lgr5-positive cells per gland, 2.3 cells, the estimation of N was 4.2 cells, which is different from the above reported estimation of $N = 5$ cells when the

initial clone size was assumed to have a Poisson distribution with and average value of 2.3 cells.

2. Proliferation and differentiation models (population dynamics)

2.1. Birth-Death stochastic model to quantify population kinetics of Lgr5-positive cells

Proliferation and differentiation of Lgr5-positive cells was modelled as a birth death stochastic process. The forward differential stochastic equations are [5]

$$\frac{dP_0}{dt} = \delta P_1$$
$$\frac{dP_k}{dt} = (k-1)\mu P_{k-1} - k(\mu+\delta)P_k + (k+1)\delta P_{k+1} \qquad k \geq 1 \qquad \text{(S16)}$$

With $P_k = \mathrm{P}\{L^+(t) = k \mid L^+(0) = i\}$ where $L^+(t)$ is the number of Lgr5-positive cells labelled with the same colour per gland at time t and $L^+(0)$ is this number at time 0; μ and δ denote the specific Lgr5-positive cell population birth and death –or differentiation- rate, respectively.

For the particular case when at the beginning there is one single labelled Lgr5-positive cell per gland, P{ $L^+(0) = 1$}=1 and P{ $L^+(0) = k$}=0 for any k different from 1, and if $\delta \neq \mu$, Equations (S16) have solution [5]:

$$P_0(t) = \delta\sigma$$
$$P_k(t) = (1-\mu\sigma)(1-\delta\sigma)(\mu\sigma)^{k-1} \qquad k \geq 1 \qquad \text{(S17)}$$
$$\sigma = \frac{1-e^{(\mu-\delta)t}}{\delta - \mu e^{(\mu-\delta)t}}$$

If $\delta = \mu$. Equations (S17) have to be replaced by [5]

$$P_0 = \frac{\mu t}{1+\mu t}$$
$$P_k = \frac{(\mu t)^{k-1}}{(1+\mu t)^{k+1}} \qquad k \geq 1 \qquad \text{(S18)}$$

2.1.1 Parameters estimation

The parameters δ and μ were estimated by MCMC methods. The chosen distribution function to construct the likelihood of the data was a multinomial with parameters $P_0(t)..P_n(t)$ which are described in Equations (S17) and (S18)

$$\mathrm{L}(\mathrm{Y} \mid \mu, \delta) = \prod_{i=1}^{m} \mathrm{Multinomial}(\mathrm{y}_0^i..\mathrm{y}_n^i; P_0(t_i)..P_n(t_i) \mid \mu, \delta)$$

Where y_k^i, is the observed number of clones with k Lgr5-positive cells labelled at the *i-th* sampling time; k = 0 .. n, with n the maximum observed number Lgr5-positive cells labelled per gland; t_i is the time at which the *i-th* sample is taken; m is the number of sampling times.

The selected non-informative prior distributions for δ and μ were:

$\pi(\mu) \sim \mathrm{Gamma}(g_1 = 0.01, g_2 = 0.01)$

$\pi(\delta) \sim \mathrm{Gamma}(g_1 = 0.01, g_2 = 0.01)$

where E [.] = g_1 / g_2 and Var [.] = g_1 / g_2^2

Fitting diagnosis and posterior distribution were analysed for δ, μ and $\mu - \delta$.

2.2 Compartmental model to quantify the population kinetics of Lgr5-negative cells.

The number of fluorescent Lgr5-positive, L^+, and Lgr5-negative, L^-, cells labelled with the same Rosa derived-colour per gland was modelled with the following system of linear differential equations.

$$\frac{dL^+}{dt} = (\mu - \delta)L^+$$
$$\frac{dL^-}{dt} = \delta L^+ + \eta L^- \qquad \text{(S19)}$$

Where μ and δ are the proliferation and differentiation rate of Lgr5-positive cells as described above and η is the proliferation rate of Lgr5-negative cells. We assumed that differentiation was associated with the loss of Lgr5 and cells did not reacquire this marker.

With initial values $L^+(0) = L_0^+$ and $L^-(0) = L_0^-$, Equations (S4) have solutions:

$$L^+ = L_0^+ e^{(\mu-\delta)t}$$
$$L^- = L_0^+ \frac{\delta}{\mu-\delta-\eta} e^{(\mu-\delta)t} + \left(L_0^- - L_0^+ \frac{\delta}{\mu-\delta-\eta} \right) e^{\eta t} \qquad \text{(S20)}$$

2.2.1 Parameters estimation

In order to estimate η, the model was fitted to data by the MCMC approach described above. The chosen distribution function to construct the likelihood of the data was the normal distribution with parameters $\ln L^-(t)$ as described in Equation (S20) and *sd*:

$$\mathrm{L}(\mathrm{X} \mid \eta, \mu, \delta, L_0^+, L_0^-, sd) = \prod_{i=1}^{m} \mathrm{Normal}(\ln x_i; \ln L^-(t_i), sd \mid \eta, \mu, \delta, L_0^+, L_0^-, sd)$$

Where ln x_i is the natural logarithm of the observed number of Lgr5-negative cells labelled with the same colour per gland at the *i-th* sampling time; t_i is the time at which the *i-th* sample is taken; *m* is the number of sampling times.

Parameters μ and δ were given a fixed value previously estimated by fitting the birth and death stochastic model with the same experimental dataset. L_0^+ was fixed to 1, which is the average initial observed number of Lgr5-positive cells labelled with the same colour per gland in this dataset.

The selected non-informative prior distributions for the rest of the parameters were as follows:

π (L_0^-) ~ Uniform (1, 50)

π (η) ~ normal (0, prec=10^{-6})

π (*sd*) ~ Gamma (g_1 = 0.001, g_2 = 0.001)

where E [*sd*] = $g_1 . g_2$ and Var [*sd*] = $g_1 . g_2^2$

3. Individual based model (IBM) for the gastric gland implementation

Spatial organization: The IBM developed for the intestinal crypt [6] was the basis to develop an IBM to analyse cell dynamics at the base of gastric gland. The spatial organization of the gastric gland was modelled as a three dimensional spiral at the base, followed by the gland neck, which was a three dimensional helix, constructed

with single cells. The cellular composition was simplified to 7 Lgr5-positive cells located at the gland base plus 50 unspecific Lgr5-negative proliferative cells completing the base and the beginning of the neck of the gland. The isthmus was not included in the model.

Cell growth and division cycle: Cells were model as two-dimensional objects. The area of proliferative cells increased linearly with time until division. The duration of the division cycles of Lgr5-positive cells had a gamma distribution with mean value equal to 11 ± 1.1 days. This value was 2 ± 0.2 for proliferative Lgr5-negative cells; the proliferative character or value of the parameters of Lgr5-negative cells did not affect the analysis of cell dynamics at the gland base. The division times of the single cells within the initial population were not synchronized but uniformly distributed along the duration of a cell cycle. Cell differentiation or loss of Lgr5 marker is associated with migration of cells above the 7 basal positions in the gland.

Cell movement: Growing cells expand in all directions and this expansion affects its position and the position of the other cells when the gland is reorganized at each time interval. Cell displacement takes place at the time of cell division. The five Lgr5-positive cells located at the base of the gland were considered the effective cells giving rise to monoclonal crypts and therefore they could replace each other and move across the gland base. After division of one of these cells, the cell to be replaced was either the cell located in the ring immediately above with probability 1-*P* or one of the other effective Lgr5-positve cells with probability *P* (Figure 4A). Two scenarios were implemented for the spatial replacement of these cells (Figure 4A). In the first scenario i) cell replacement affected either the cell on the left or on the right with the same probability, i.e cells move along a one-dimensional ring; in scenario ii) cell replacement takes place in two dimensions, so that any of the other effective Lgr5 cells have the same chance to be replaced. In both scenarios, the just replaced cell could either move upwards with probability 1-*P* or could replace one of the other effective Lgr5-positive cells. A necessary restriction is that one cell cannot be affected twice in the same division event.

Cells located out of the 5 basal effective positions move always upwards to the ring immediately above. Vertical cell displacement spreads upwards until the last ring modelled in the gland.

Supplementary Material References

1 Lopez-Garcia, C., Klein, A. M., Simons, B. D. & Winton, D. J. Intestinal stem cell replacement follows a pattern of neutral drift. *Science* **330**, 822-825, doi:10.1126/science.1196236 (2010).

2 Snippert, H. J. *et al.* Intestinal crypt homeostasis results from neutral competition between symmetrically dividing Lgr5 stem cells. *Cell* **143**, 134-144, doi:10.1016/j.cell.2010.09.016 (2010).

3 Vermeulen, L. *et al.* Defining stem cell dynamics in models of intestinal tumor initiation. *Science* **342**, 995-998, doi:10.1126/science.1243148 (2013).

4 Kozar, S. *et al.* Continuous clonal labeling reveals small numbers of functional stem cells in intestinal crypts and adenomas. *Cell stem cell* **13**, 626-633, doi:10.1016/j.stem.2013.08.001 (2013).

5 Feller, W. in *Proceedings of the First Berkeley Symposium on Mathematical Statistics and Probability. August 13-18, 1945 and January 27-29, 1946.* (ed J. Neyman) 403-432 (University of California Press, 1949).

6 Pin, C., Watson, A. J. & Carding, S. R. Modelling the spatio-temporal cell dynamics reveals novel insights on cell differentiation and proliferation in the small intestinal crypt. *Plos One* **7**, e37115, doi:10.1371/journal.pone.0037115 (2012).

7 Leushacke, M., Ng, A., Galle, J., Loeffler, M. & Barker, N. Lgr5(+) gastric stem cells divide symmetrically to effect epithelial homeostasis in the pylorus. *Cell Rep* **5**, 349-356, doi:10.1016/j.celrep.2013.09.025 (2013).

Greater Curvature

Number of labelled Lgr5+ cells (columns); Number Lgr5+ cells (rows)

Day 0	0	1	2	3	4	5	6	7	8	9	10	>10
0												
1												
2												
3												
4			2									
5		1	4	4	4	3						
6	1			6	6	3	1					
7		1		2	2	2	3	1				
8			1	1	4	2		1	1			
9					1	2	1	1	1			
10						1	1	1		1		
>10									1		1	1

Day 5	0	1	2	3	4	5	6	7	8	9	10	>10
0												
1												
2												
3												
4			1	2	1							
5		1	1	7	4	2						
6			1	4	6	6	2					
7				5	2	5	5	1				
8				1	2	2	2	4	1			
9				1	1			3	1			
10						1	1	1				
>10										1	1	

Day 12	0	1	2	3	4	5	6	7	8	9	10	>10
0												
1												
2												
3		2	1									
4			1	2	4							
5	6	3	1	3	3							
6	3	2	2	13	2	1	3					
7		2	1	3	5	3	4	1				
8					4	5	1	2				
9		1	1	1	1	3	7	2				
10		1	1	1		2	1	5	1			
>10			1			1	1	3	1	4	1	

Day 40	0	1	2	3	4	5	6	7	8	9	10	>10
0												
1												
2	4		1									
3	2	1	1	1								
4	6	1	4	5								
5	8	7	5	2	5	3						
6	5	4	8	7	4	1	2					
7	9	5	4	7	4	6	2	4				
8	5	4	4		4	5	4	5	4			
9	2	4	3	1	4	2	4	2				
10		1	1	2	2	5	2	1	1		1	
>10					2	4	3	2	6	4	4	

Day 59	0	1	2	3	4	5	6	7	8	9	10	>10
0												
1	1											
2	2	1	1									
3	8	2	3	3								
4	12	7	8	7	9							
5	12	3	9	8	8	11						
6	7	9	8	8	3	5	14					
7			6	10	2	6	4	10				
8	1		1	2	4	4	2	1				
9				1		2	1	1	2	2		
10					1			1		2	1	
>10				1		1					1	1

Lesser Curvature

Number of labelled Lgr5+ cells (columns); Number Lgr5+ cells (rows)

Day 0	0	1	2	3	4	5	6	7	8	9	10	>10
0												
1												
2												
3		1										
4		1	2	1	1							
5	1	2	3		1	1						
6		3	2	4	6							
7		1	2		3	1	2					
8			3	2	3	2						
9			1			1	3	1				
10				1		1	2	2				
>10				1						1	2	

Day 5	0	1	2	3	4	5	6	7	8	9	10	>10
0												
1												
2			1									
3			1									
4	2		3	1								
5	1		2	3	1	1						
6				2	5		2					
7				3	2	8		2				
8	1			2	3	3	1	2				
9			1	2	4	1	1	1		1		
10					1		1	1		1		
>10						2		3	2	1	1	

Day 12	0	1	2	3	4	5	6	7	8	9	10	>10
0												
1												
2	1											
3		1										
4	1	3	2	2								
5		1	5	3	6							
6	2	2	4	2	3	1						
7		2	1	3	1	1		1				
8	3	2	3	5	4	2	3					
9			1		2		4		2	1		
10		1	2	1		4	2		1			
>10	1			4	2		3	2	2	2		1

Day 40	0	1	2	3	4	5	6	7	8	9	10	>10
0												
1												
2												
3	2	1	1									
4	2	1	3	1								
5		2	2	1	1	1						
6	5	2					1					
7	3	3	5	1	2		1	3				
8		1										
9	3		1	2		2	1					
10	3			1				2	2		1	
>10	2	2	3	2	2	1	2	2	1	1		

Day 59	0	1	2	3	4	5	6	7	8	9	10	>10
0												
1	1											
2	6		1									
3	10	1	2									
4	8	2		4	2							
5	5	5	3	3	1	2						
6	12	5	7	5	4	5	4					
7	1	2	3	4		3	2	2				
8		1	1	2	2	1	3	1	1			
9		1		1	1	2	1					
10	1				1	1	1	1	1			
>10	1		1	1	2		1	1	2			

Supplementary Figure 1. Cell counts in tracing studies of Lgr5-positive cells in pyloric glands located on the greater (left hand side matrices) and lesser stomach curvature (right hand side matrices). Matrices indicate the absolute number of glands scored for each given count of Lgr5-positive cells and of labelled Lgr5-positive cells after tamoxifen activation. Cell counts were carried out on 69, 79, 123, 226 and 240 pyloric glands on the greater curvature and on 64, 76, 108, 81, and 148 pyloric glands on the greater curvature at 0, 5, 12, 40 and 59 days, respectively, after allele recombination due to Tamoxifen action.

A

Simulated datasets			Fitting results		
			Assumed	Estimated	
$S(0)$	N	λ	$S(0)$	N	λ
1	5	0.1	1	5	0.104
2	5	0.1	2	5	0.103
			1	4	0.0683
3	5	0.1	3	5	0.103
			1	2	0.0312
4	5	0.1	4	5	0.104
			1	2	0.0287

B

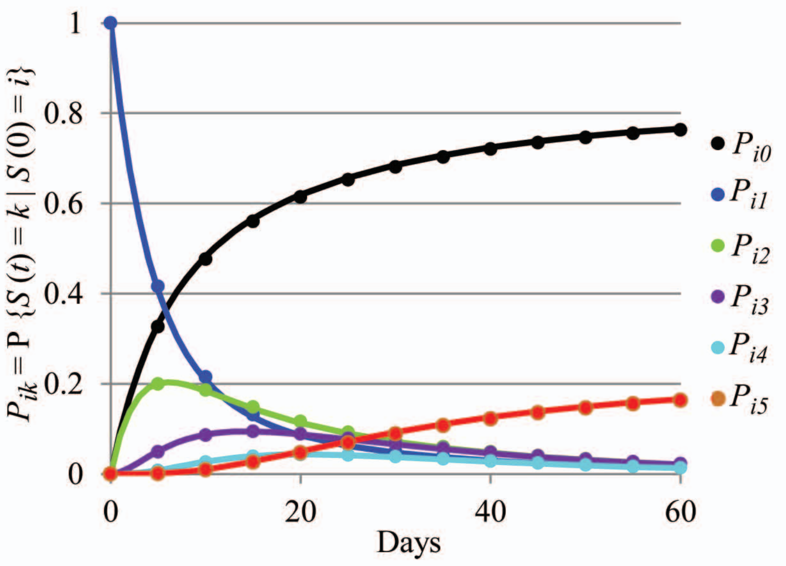

C

D

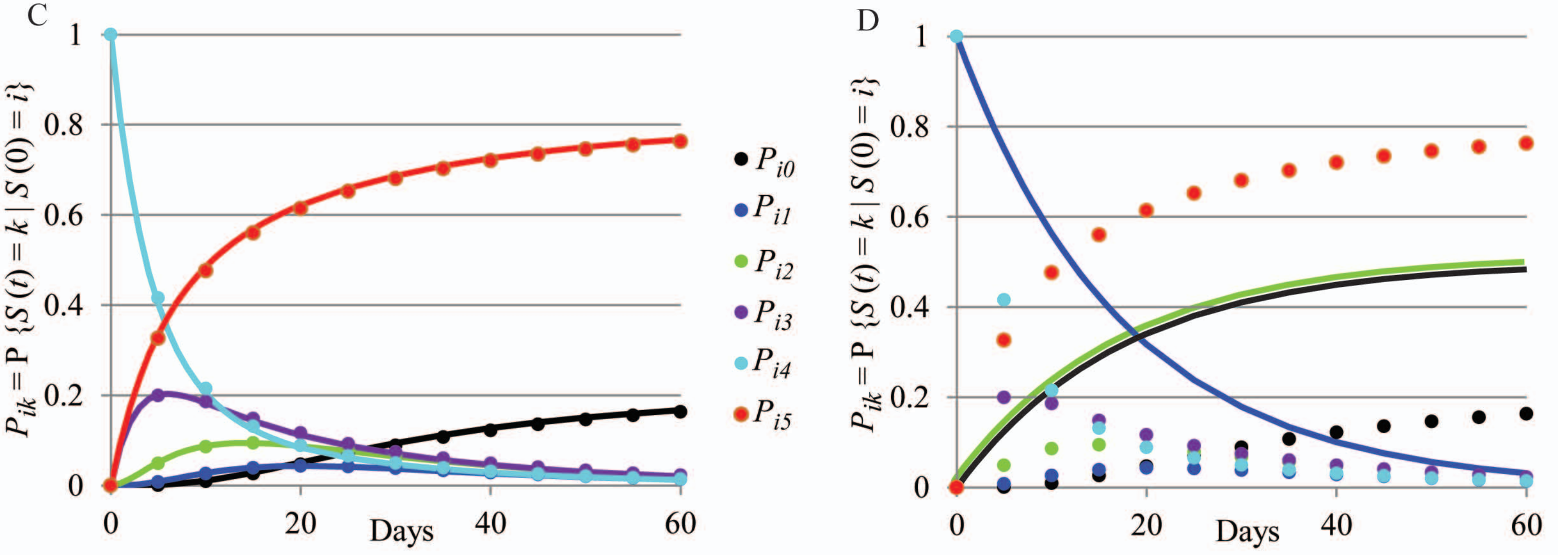

E

Initial clone size	N
$PP(\alpha = 2.3)$	5
1	2
2	4
2.3	4.20
3	5.30
4	7.10

Supplementary Figure 2. Effect of initial clone size on the fitting results of clonal expansion models. A) Table shows that the fitted values of the parameters λ and N, were similar to the true values used to generate the data only when the initial clone size assumed in the model for $P_{ij}(t) = P\{S(t) = j \mid S(0) = i\}$ in Equation (S5) was equal to that used for data generation; B) Generated frequencies (dots) and fitted probabilities (lines) for $P_{ij}(t)$ assuming 1 initial cell per clone and C) assuming 4 initial cells per clone; D) Data points (dots) were generated with 4 initial cells per clone while the fitted model (lines) for $P_{ij}(t)$ assumes that the initial clone size is equal to 1. All data in A-D) was generated using Equation (S5) with a clone replacement rate, λ, was 0.1 day^{-1} and the number per cells in the ring at the base of the gland, N, was 5 in all simulations. E) Table shows the estimated values of the parameter N by fitting the limiting probability of extinction, Π_{ij} in Equation (S2) to the dataset in Figure 2A but assuming several values/distributions for the initial clone size.

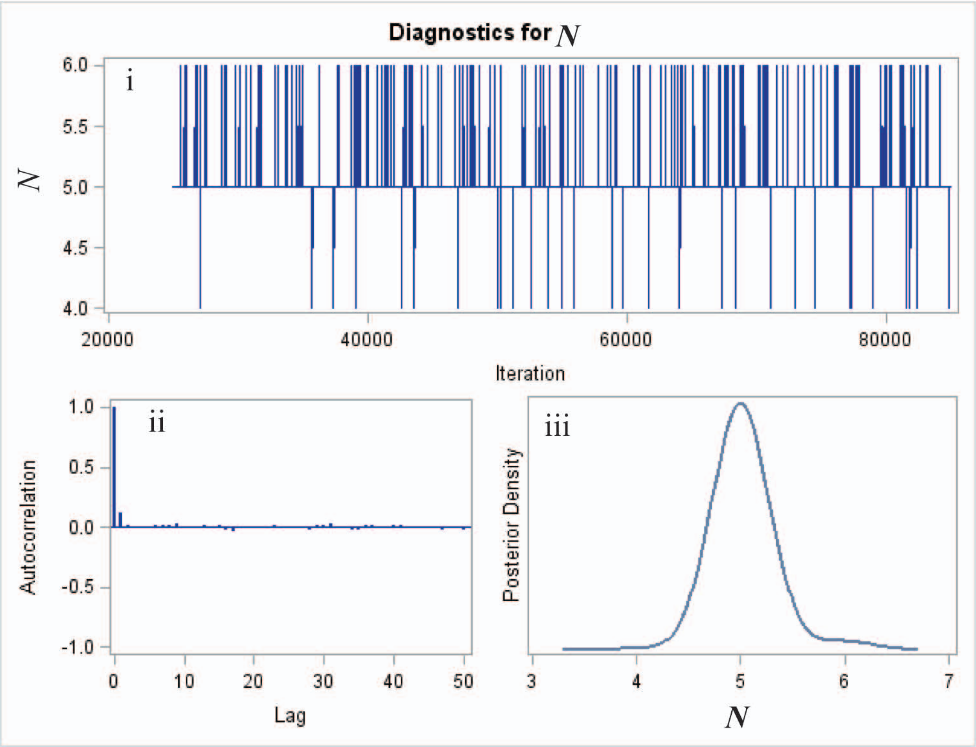

iv		Parameter *N*
**Posterior Summaries**		
**n**		6000
**Mean**		5.028
**Standard deviation**		0.1947
**Percentiles**	**25%**	5
	**50%**	5
	**75%**	5
**Posterior Intervals (Alpha = 0.05)**		
**Equal-Tail Interval**		5
		6
**HPD Interval**		5
		5

Supplementary Figure 3. MCMC output result from fitting the probability of extinction to the proportion of glands with non-labelled Lgr5-positive cells at late sampling times showing limiting behaviour in a previously reported dataset [7] derived from a long term lineage tracing 4 colours experiment activated with a relatively high dose of tamoxifen. Plots i show the traces for parameter *N* or value estimated in each step of the MCMC. Plots ii are the autocorrelation functions for the estimates of parameter *N*. Plots iii show the posterior distributions; Tables iv show the mean, error, percentiles and credible intervals estimated for each parameter.

A

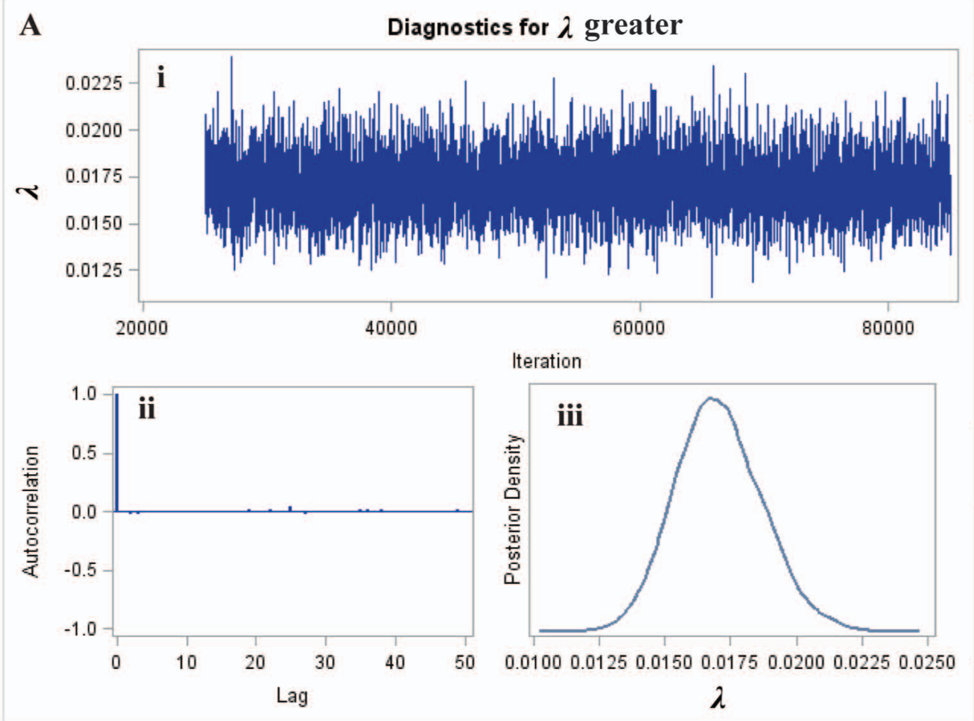

iv		Parameter λ
**Posterior Summaries**		
**n**		6000
**Mean**		0.0171
**Standard deviation**		0.00164
**Percentiles**	**25%**	0.0159
	**50%**	0.017
	**75%**	0.0181
**Posterior Intervals (Alpha = 0.05)**		
**Equal-Tail Interval**		0.014
		0.0205
**HPD Interval**		0.0139
		0.0202

B

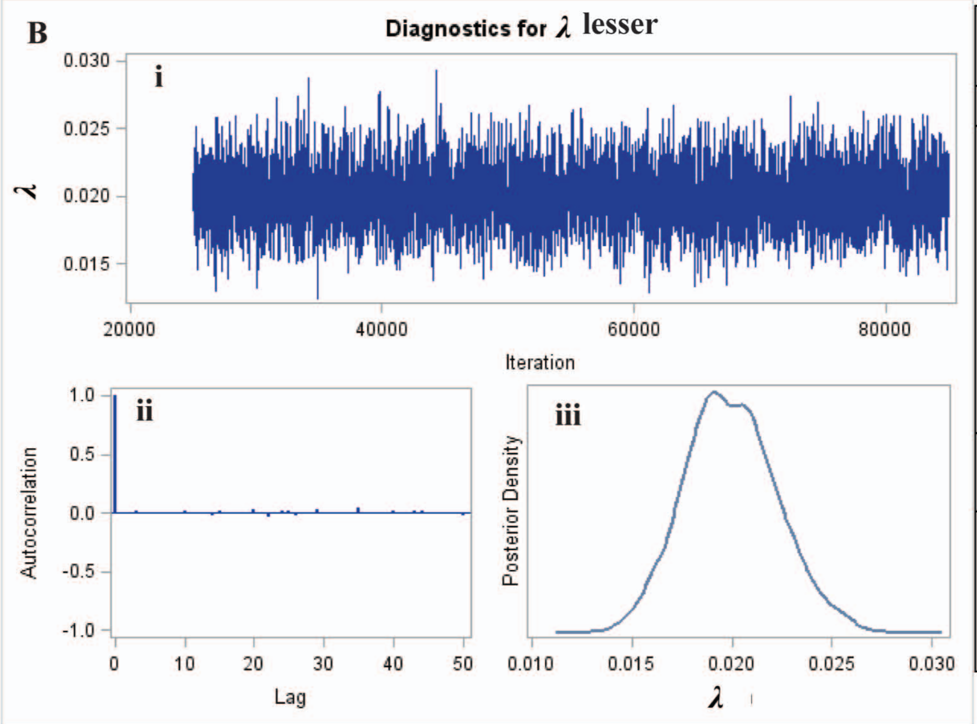

iv		Parameter λ
**Posterior Summaries**		
**n**		6000
**Mean**		0.02
**Standard deviation**		0.00229
**Percentiles**	**25%**	0.0184
	**50%**	0.0199
	**75%**	0.0215
**Posterior Intervals (Alpha = 0.05)**		
**Equal-Tail Interval**		0.0157
		0.0247
**HPD Interval**		0.0155
		0.0245

C

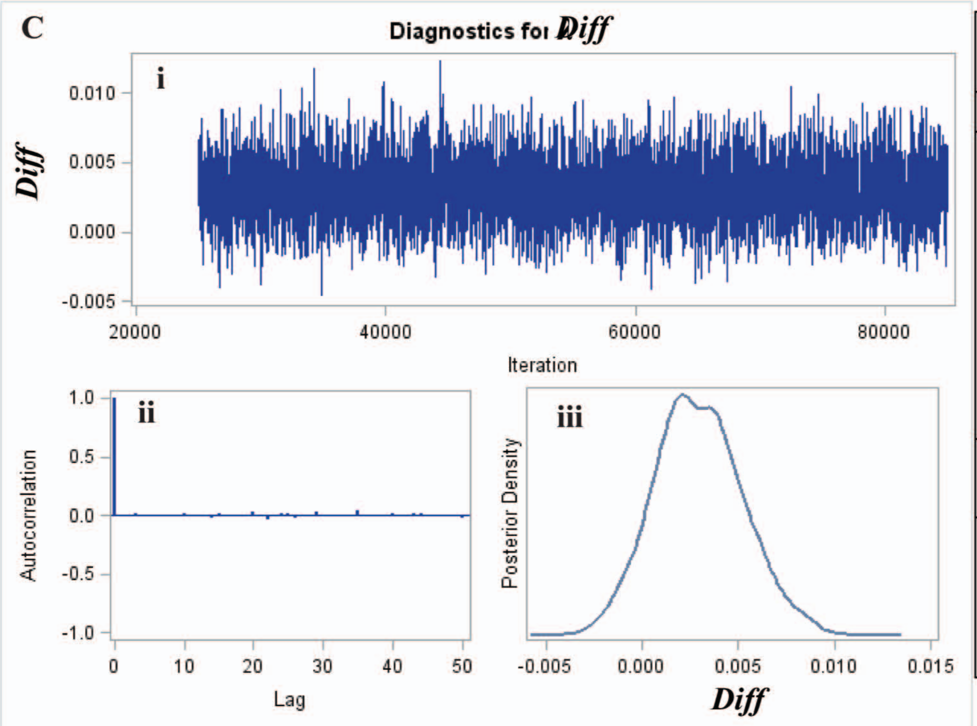

iv		Parameter *Diff*
**Posterior Summaries**		
**n**		6000
**Mean**		0.00297
**Standard deviation**		0.00229
**Percentiles**	**25%**	0.00137
	**50%**	0.00288
	**75%**	0.00449
**Posterior Intervals (Alpha = 0.05)**		
**Equal-Tail Interval**		-0.0013
		0.00771
**HPD Interval**		-0.0015
		0.0075

Supplementary Figure 4. MCMC output result to estimate the clone replacement rate at the gland base by fitting the clonal expansion model to newly generated datasets in this work by bottom view confocal microscopy observation of pyloric glands located on the greater (A) and on the lesser (B) stomach curvatures. The MCMC output result is also shown for the difference between the replacement rates in glands from the lesser and from the greater stomach curvature (C). Plots i show the traces for parameter λ or value estimated in each step of the MCMC. Plots ii are the autocorrelation functions for the estimates of parameter λ. Plots iii show the posterior distributions; Tables iv show the mean, error, percentiles and credible intervals estimated for each parameter.

A

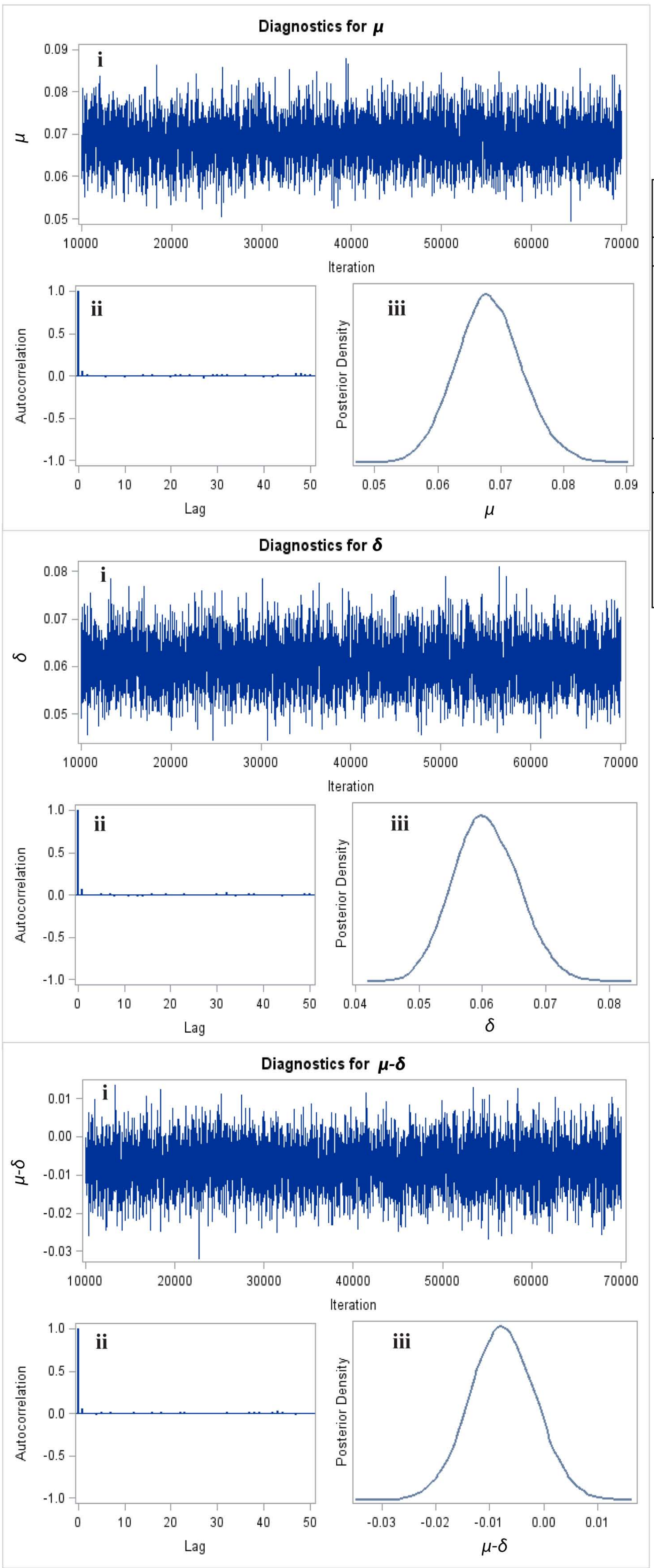

iv	Parameter		
	**μ**	**δ**	**μ-δ**
**Posterior Summaries**			
**N**	6000	6000	6000
**Mean**	0.0682	0.0606	-0.0076
**Standard deviation**	0.0052	0.0051	0.006
**25%**	0.0647	0.0571	-0.0117
**Percentiles 50%**	0.0681	0.0605	-0.0077
**75%**	0.0716	0.0641	-0.0035
**Posterior Intervals (Alpha = 0.05)**			
**Equal-Tail Interval**	0.0582<br>0.0789	0.051<br>0.0709	-0.0195<br>0.0042
**HPD Interval**	0.0581<br>0.0787	0.051<br>0.0709	-0.0194<br>0.0042

B

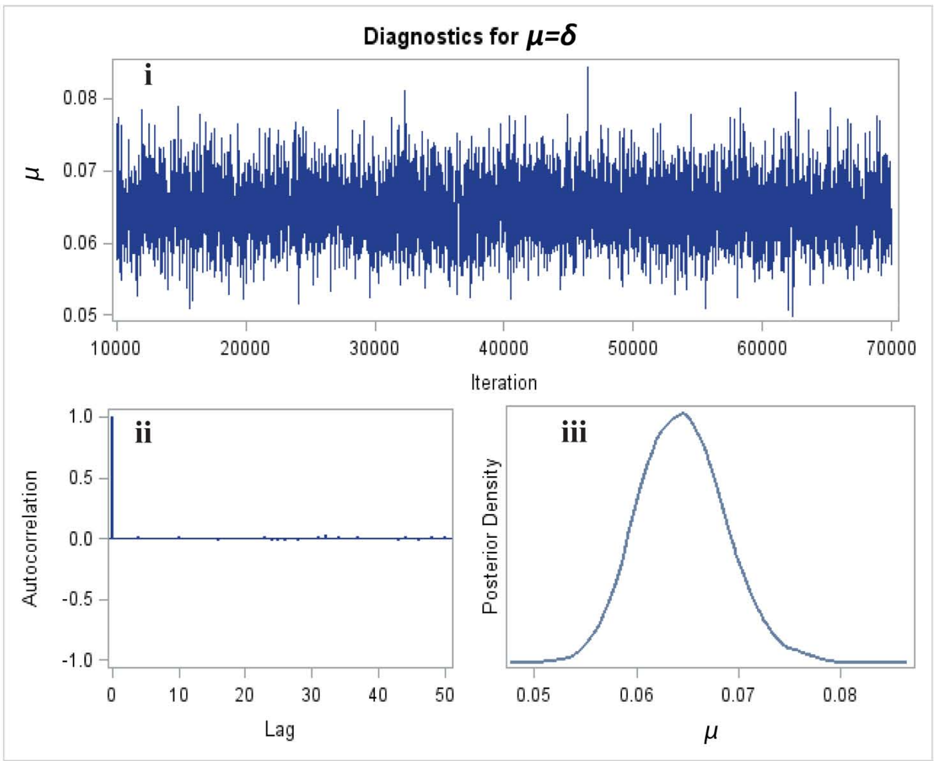

iv	Parameter μ=δ
**Posterior Summaries**	
**N**	6000
**Mean**	0.0645
**Standard deviation**	0.00425
**25%**	0.0615
**Percentiles 50%**	0.0644
**75%**	0.0673
**Posterior Intervals (Alpha = 0.05)**	
**Equal-Tail Interval**	0.0566<br>0.0732
**HPD Interval**	0.056<br>0.0725

Supplementary Figure 5. MCMC output result from fitting the birth death model with different birth and death rates (A) and for the particular case when the birth rate is equal to the death rate (B) to a short term dataset activated with a low dose of tamoxifen previously reported [7]. Plots i show the traces for each parameter or value estimated in each step of the MCMC. Plots ii are the autocorrelation functions for the estimates of each parameter. Plots iii show the posterior distributions; Tables iv show the mean, error, percentiles and credible intervals estimated for each parameter.

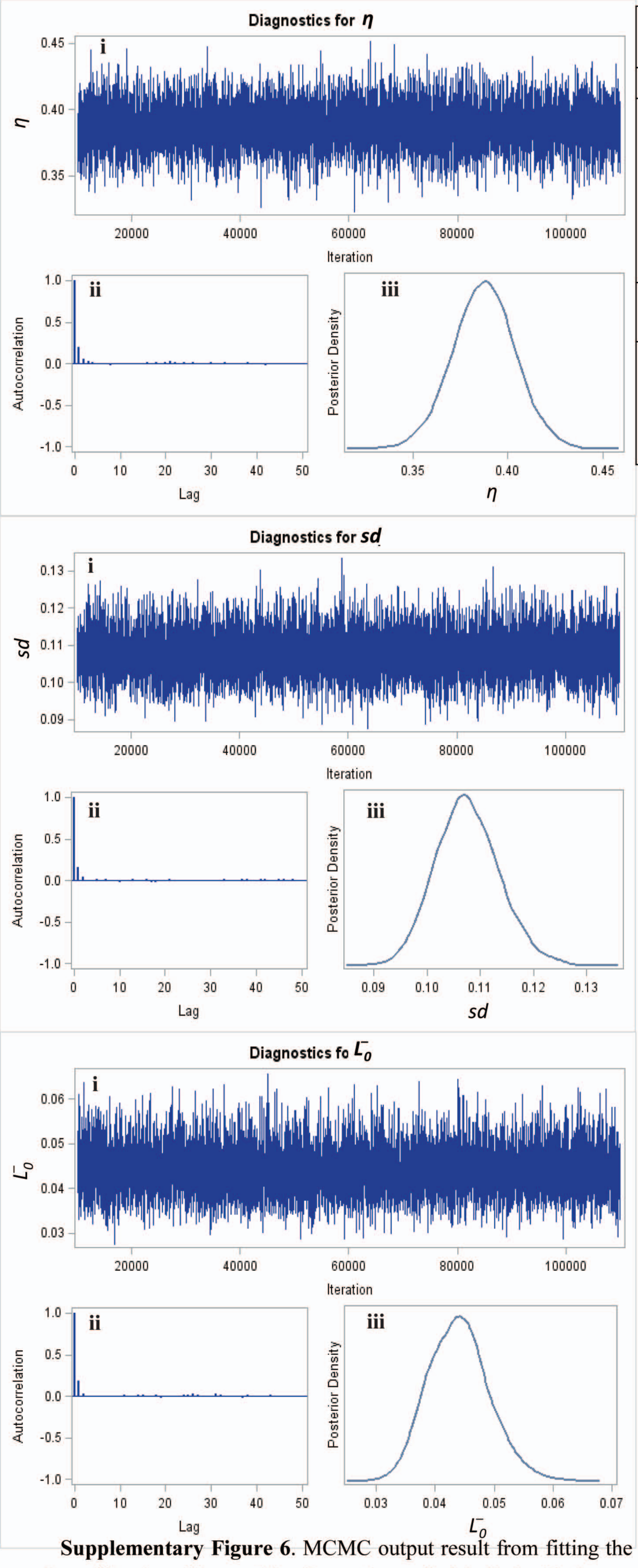

iv

		Parameter		
		η	sd	$\bar{L}_0$
**Posterior Summaries**				
	**N**	10000	10000	10000
	**Mean**	0.3876	0.1077	0.0441
	**Standard deviation**	0.0164	0.00598	0.00519
**Percentiles**	**25%**	0.3767	0.1035	0.0404
	**50%**	0.3877	0.1074	0.0439
	**75%**	0.3985	0.1115	0.0473
**Posterior Intervals (Alpha = 0.05)**				
**Equal-Tail Interval**		0.3546	0.0964	0.0346
		0.42	0.1201	0.055
**HPD Interval**		0.3561	0.0958	0.0339
		0.4212	0.1193	0.0541

Supplementary Figure 6. MCMC output result from fitting the compartmental model describing the number of Lgr5-positive and Lgr5-negative cells labelled with the same colour per gland over time. Plots i show the traces for each parameter or value estimated in each step of the MCMC. Plots ii are the autocorrelation functions for the estimates of each parameter. Plots iii show the posterior distributions; Tables iv show the mean, error, percentiles and credible intervals estimated for each parameter.

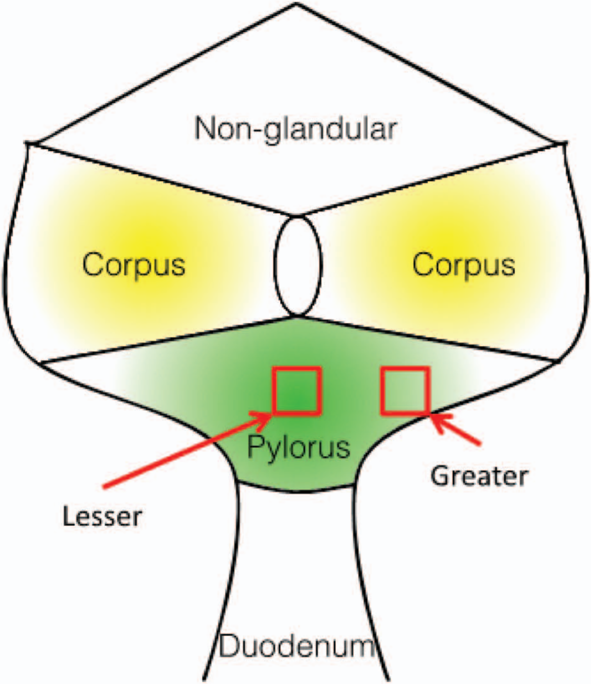

Supplementary Figure 7. Cartoon of the lumen side of the gastric mucosa in a sliced open stomach. Red squares represent the greater and lesser curvature regions of the pyloric epithelium imaged with confocal microscopy in this work in this work.
